# Pharmacokinetics of Isoniazid, Pyrazinamide, and Ethambutol in Newly Diagnosed Pulmonary TB Patients in Tanzania

**DOI:** 10.1371/journal.pone.0141002

**Published:** 2015-10-26

**Authors:** Paolo Denti, Kidola Jeremiah, Emmanuel Chigutsa, Daniel Faurholt-Jepsen, George PrayGod, Nyagosya Range, Sandra Castel, Lubbe Wiesner, Christian Munch Hagen, Michael Christiansen, John Changalucha, Helen McIlleron, Henrik Friis, Aase Bengaard Andersen

**Affiliations:** 1 Division of Clinical Pharmacology, Department of Medicine, University of Cape Town, Cape Town, South Africa; 2 Department of Infectious Diseases, Odense University Hospital, Odense, Denmark; 3 National Institute for Medical Research, Mwanza Medical Research Centre, Mwanza, Tanzania; 4 Department of Nutrition, Exercise and Sports, Faculty of Science, University of Copenhagen, Copenhagen, Denmark; 5 National Institute for Medical Research, Muhimbili Research Centre, Dar Es Salaam, Tanzania; 6 Department for Congenital Disorders, Statens Serum Institut, Copenhagen, Denmark; 7 Department of Infectious Diseases, Rigshospitalet, Copenhagen, Denmark; Public Health Research Institute at RBHS, UNITED STATES

## Abstract

Exposure to lower-than-therapeutic levels of anti-tuberculosis drugs is likely to cause selection of resistant strains of *Mycobacterium tuberculosis* and treatment failure. The first-line anti-tuberculosis (TB) regimen consists of rifampicin, isoniazid, pyrazinamide, and ethambutol, and correct management reduces risk of TB relapse and development of drug resistance. In this study we aimed to investigate the effect of standard of care plus nutritional supplementation versus standard care on the pharmacokinetics of isoniazid, pyrazinamide and ethambutol among sputum smear positive TB patients with and without HIV. In a clinical trial in 100 Tanzanian TB patients, with or without HIV infection, drug concentrations were determined at 1 week and 2 months post initiation of anti-TB medication. Data was analysed using population pharmacokinetic modelling. The effect of body size was described using allometric scaling, and the effects of nutritional supplementation, HIV, age, sex, CD4+ count, weight-adjusted dose, NAT2 genotype, and time on TB treatment were investigated. The kinetics of all drugs was well characterised using first-order elimination and transit compartment absorption, with isoniazid and ethambutol described by two-compartment disposition models, and pyrazinamide by a one-compartment model. Patients with a slow NAT2 genotype had higher isoniazid exposure and a lower estimate of oral clearance (15.5 L/h) than rapid/intermediate NAT2 genotype (26.1 L/h). Pyrazinamide clearance had an estimated typical value of 3.32 L/h, and it was found to increase with time on treatment, with a 16.3% increase after the first 2 months of anti-TB treatment. The typical clearance of ethambutol was estimated to be 40.7 L/h, and was found to decrease with age, at a rate of 1.41% per year. Neither HIV status nor nutritional supplementations were found to affect the pharmacokinetics of these drugs in our cohort of patients.

## Introduction

The aim of anti-tuberculosis (TB) treatment is to provide a safe, effective, and fast acting therapy [1]. Isoniazid, pyrazinamide, and ethambutol constitute important companion drugs used in a standard first-line short-course regimen together with rifampicin [2] and are believed to eradicate aerobic, anaerobic, microaerophilic, and drug tolerant persisting bacteria [3]. While isoniazid and pyrazinamide have bactericidal activity against *M*. *tuberculosis*, ethambutol is considered a bacteriostatic drug, though it may have bactericidal activity when given in higher doses [2]. Treatment success rates of 88% were reported in Tanzania in 2011 using this regimen, thereby meeting the 85% target set by the World Health Assembly in 1993 [4, 5]. However, multidrug-resistance (MDR-TB) is emerging (1.1% of newly diagnosed TB cases in Tanzania are MDR-TB) and may over time threat the standard first-line regimen [4, 5]. Previous studies have shown that low plasma anti-TB drug concentrations may result in treatment failure [6, 7] and low plasma concentrations of rifampicin and isoniazid have been associated with MDR-TB [8, 9]. Wide variability is reported in the pharmacokinetics (PK) of isoniazid, pyrazinamide, and ethambutol [10–12], with factors such as age and HIV status and antiretroviral treatment (ART) possibly affecting TB drug concentrations [12–17]. Furthermore, malnutrition also seems to affect drug exposure by decreasing total clearance and increasing plasma half-life [18]. Nutritional rehabilitation of children with kwashiorkor has been reported to enhance isoniazid clearance [19], but the influence of administering nutritional supplementation to adult TB patients is unclear. We therefore conducted a randomized clinical trial in Mwanza, Tanzania to examine the effect of nutritional supplementation on the pharmacokinetics of first-line anti-TB drugs in a cohort of pulmonary TB patients with or without HIV. We recently reported the positive effect on a nutritional supplementation on rifampicin exposure in the HIV co-infected patients (all ART naïve) [20]. In this analysis we aimed to investigate the effect of standard of care plus nutritional supplementation vs. standard care on PK of isoniazid, pyrazinamide, and ethambutol among sputum smear positive TB patients with and without HIV. We also explored the effect of other covariates, including NAT2 genotype on the PK of isoniazid.

## Materials and Methods

### Ethics Statement

Ethical permission to conduct the study was granted by the Medical Research Coordinating Committee (MRCC) of the National Institute for Medical Research (NIMR) in Tanzania. Oral and written information were provided in Swahili to all participants prior to obtaining informed oral and written consent. Written consent was obtained from parents/legal guardians of participants aged 15–17 years.

### Study design, setting, and participants

The study was an open-label randomized clinical trial (ControlledTrials.com: ISRCTN 16552219) among 100 sputum smear positive TB patients, and details about the protocol are available as supplementary information) (S1 Protocol). The study was conducted in the city of Mwanza, Tanzania between September 2010 and August 2011. Mwanza is the second largest city in the country and is the region with the second highest number of TB case notification (9.3%) after Dar es Salaam (21.9%) [5]. The study recruited newly diagnosed sputum positive pulmonary TB patients aged 15 years or above. HIV-infected patients on ART, pregnant women, critically ill patients not likely to survive > 48 hours, and non-residents of Mwanza City were excluded.

### TB medication and intervention

The TB patients were administered TB medication according to the National Tuberculosis and Leprosy Programme (NTLP) treatment guidelines [5], and those found co-infected with HIV were managed according to National guidelines for the management of HIV and AIDS policy [21]. The anti–TB drugs prescribed were formulated in fixed–dose combination (FDC) tablets containing isoniazid (75 mg), rifampicin (150 mg), pyrazinamide (400 mg), and ethambutol (275 mg) (Sandoz Pvt Ltd, India). Dosing was adjusted based on body weight: 3 tablets for patients weighing up to 50 kg, and 4 tablets for those weighing more than 50 kg. The patients were randomized to either receive or not receive nutritional supplementation in the form of biscuits (Compact A/S, Bergen, Norway) containing high-energy (1000 kcal) and vitamin/minerals according to trial protocol [20]. A complete list of nutrients content is shown in **Table 1.**

**Table 1:** Nutrients composition of the intervention used in the trial.

1 daily biscuit with the following micro nutrients^a^
Vitamin A(5000IU)
Vitamin B_1_ (20mg)
Vitamin B^2^ (20mg)
Vitamin B_6_ (25mg)
Vitamin B_12_ (50mg)
Folic acid (0.8mg)
Niacin (40mg)
Vitamin C (200mg)
Vitamin E (60mg)
Vitamin D_3_ (200iu)
Selenium 0.2 mg
Copper (5mg)
Zinc (30mg)
Plus 4 additional daily biscuit with energy-protein

^a^Duration of intervention 60 days

### Data collection

A standardised questionnaire was used to solicit demographic characteristics, previous TB history, and use of alcohol. Anthropometric measurements including weight and height were obtained at each visit. All participants had a chest x-ray taken at recruitment and two independent radiographers confirmed abnormalities.

### Laboratory analyses

At recruitment, venous blood samples were collected. CD4+ lymphocyte count was analysed using a Coulter® Epics XL-MCL™ Flow Cytometer (Beckman Coulter, Brea, CA). Haemoglobin level and white blood cell counts were analysed using haematological Coulter AcT 5 diff, (Beckman Coulter, Brea, CA). HIV status was determined on two rapid tests done in parallel (SD Bioline HIV-1/2 3.0, Standard Diagnostics Inc., Kyonggi-do, South Korea; Determine HIV-1/HIV-2, Inverness Medical Innovations Inc., Delaware, USA). Discordant HIV test results were resolved using HIV UNIFORM II ELISA (Organon Teknia Ltd, Boxtel, Netherlands),

### Pharmacokinetic plasma sample collection, processing and analysis

Patients were scheduled for plasma sample collection on two occasions: at one week and two months post-initiation of anti-TB medication. One day before blood sampling patients were instructed to fast overnight, and on the morning of the PK visit, the study nurse administered the anti-TB drugs according to body weight. Whole blood was collected in 5 mL of lithium heparin tubes at 2, 4, and 6 hours post-dose. Samples were immediately centrifuged at 3000 rpm for 10 min to separate the plasma and transferred to -80°C within 30 minutes. The plasma samples were then transported in dry ice to the Division of Clinical Pharmacology, University of Cape Town, South Africa, for determination of isoniazid, pyrazinamide, and ethambutol concentrations using validated tandem mass spectrometry high-performance liquid chromatography (LC-MS/MS) methods. An AB Sciex API mass spectrometer was operated in the multiple reactions monitoring (MRM) mode. The assays were validated over the concentration range of 0.112 to 26 mg/L for isoniazid, 0.203 to 81.1 mg/L for pyrazinamide and 0.081 to 5.18 mg/L for ethambutol. The mean percentage accuracies during inter-day sample analysis at low, medium, and high quality control levels, respectively, were 98.2%, 99.3%, and 94.7% for isoniazid, 97.8%, 102.1%, and 100.5% for pyrazinamide, and 99%, 101.3%, and 99.6% for ethambutol. The precision coefficient of variation for determination at low, medium, and high quality control level for both pyrazinamide and ethambutol was less than 4% and for isoniazid less than 5%. Concentrations below the validation range of the assay were reported as below the lower limit of quantification (BLQ).

### DNA extraction and NAT2 analysis

Serum aliquots for N-acetyltransferase-2 (NAT2) genotype were kept under -80°C until analyzed. Genotyping was carried out at the Statens Serum Institut, Copenhagen, DK after DNA was extracted from blood using QIAamp DNA minikit (Qiagen GmbH, Hilden, Germany). An annealing temperature of 60°C was used in all polymerase chain reactions (PCR). The PCR products were sequenced using BigDye Terminator v1.1 Cycle Resequencing (*ABI*), and analyzed on an ABI3730 DNA Analyzer. The resulting sequences were compared to NCBI accession no. NG_012246.1 *N*-acetyltransferase-2 (NAT2) using Sequencer 5.0 software (Gene Codes, Ann Arbor, USA). NAT2 haplotypes were based on dbSNP IDs: c.282C>T (rs1041983), c.341T>C (rs1801280), c.481C>T (rs1799929), c.590G>A (rs1799930), c.803A>G (rs1208) and c.857G>A (rs1799931) and the acetylator phenotype was inferred using NAT2PRED (http://nat2pred.rit.albany.edu/).

### Nonlinear mixed-effects modelling analysis

Nonlinear mixed-effects modelling was employed to interpret the data with the software NONMEM 7.3 [22], and the algorithm First-Order Conditional Estimation with eta-epsilon interaction. Pirana, Perl-speaks-NONMEM, and xpose4 were used to aid the modelling process and prepare model diagnostics [23]. The modelling procedure was similar for all drugs, as outlined below. Several structural models were tested: one- and two-compartment disposition kinetics with first-order elimination and several approaches for absorption: first-order, lagged first-order and transit compartment absorption [24]. The statistical model assumed log-normal distribution for the between-subject and–occasion random effects, and a combined additive and proportional structure for the residual unexplained variability, with the additive component of the error bound to be at least 20% of the lower limit of quantification (LLOQ). Allometric scaling with either total body weight (WT) or fat-free mass (FFM) was applied to all clearances (CL and Q) and volumes of distribution (V_c_ and V_p_), as advocated by Anderson and Holford [25]. The effect of other covariates was tested and included in the model based on significant decreases (p<0.05) in the Objective Function Value (OFV) and physiological plausibility. Covariates tested for effects on PK parameters were: HIV co-infection, nutritional supplementation, age, sex, CD4+ lymphocyte count, daily weight-adjusted dose, and time on TB treatment. Additionally, NAT2 acetylator status was tested on isoniazid PK. The OFV, goodness of fit plots, and Visual Predictive Checks (VPC) guided model development. The robustness of the final parameter estimates was assessed with a non-parametric bootstrap. The post-hoc individual parameter estimates from the final model were used to obtain the exposure parameters C_max_ and AUC_0-24_. These individual values were calculated to provide summary values for comparison with previous studies, but they were not used. with the purpose of statistical inference, since they are dependent on the model and they are affected by statistical shrinkage (especially C_max_)[26].

## Results

**Fig 1:**
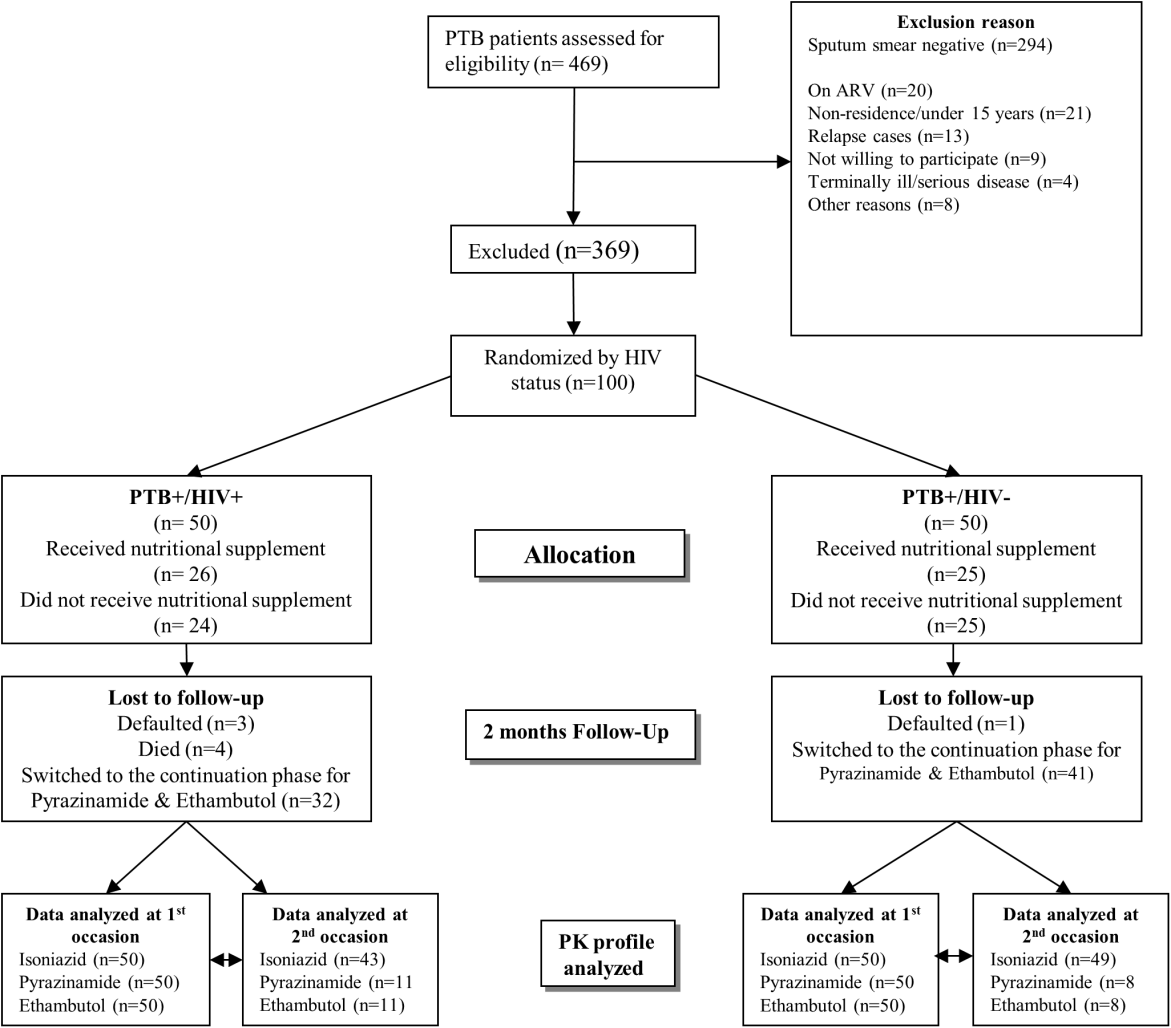
Flow chart for pulmonary TB-positive patients randomized to receive a nutritional supplement or no supplement and followed for the effects of nutritional supplementation on Isoniazid, Pyrazinamide and Ethambutal exposure at the end of the second month of intensive phase of treatment.

A total of 100 newly diagnosed pulmonary TB patients were enrolled in the study **(Fig 1)**. The sex and HIV status distributions were almost even, with 42% (n = 42) women and 50 HIV co-infected. The median (IQR) age was 35 years (29; 40) and weight was 51.9 kg (48.2; 57.3). As many as 48 subjects were classified as NAT2 slow acetylators, 48 as intermediate, and 2 as rapid acetylators, while the acetylator status of 2 subjects could not be determined. Baseline characteristics are shown in **Table 2.**

**Table 2:** Baseline characteristics of 100 pulmonary sputum smear positive patients starting TB treatment.

Characteristics	Values^a^
Age (years)	35 (29; 40)
Sex (Males)	58 (58.0)
Weight (kg)	51.9 (48.3; 57.3)
Body mass index (kg/m^2^)	18.8 (17.3; 19.9)
Haemoglobin (g/dL)^b^	111 (93; 125)
White blood cell (x 10^9^/L)^b^	6.6 (4.6; 9.1)
CD4+ count (cell/μL)^b^	375 (160; 642)
HIV infected	50 (50.0)
TB cavitations present	28 (32.2)
Fasting blood glucose (mmol/L)	6 (5.4; 6.6)
Nutritional supplementation	
No Supplementation	49 (49.0)
Received supplementation	51 (51.0)
NAT2 phenotype	
Slow	48(48.0)
Intermediate	48 (48.0)
Rapid	2 (2.0)
Unknown	2 (2.0)

^a^Data are median (IQR) or n (%).

^b^The total number of observations was not 100 due to missing values.

**Fig 2:**
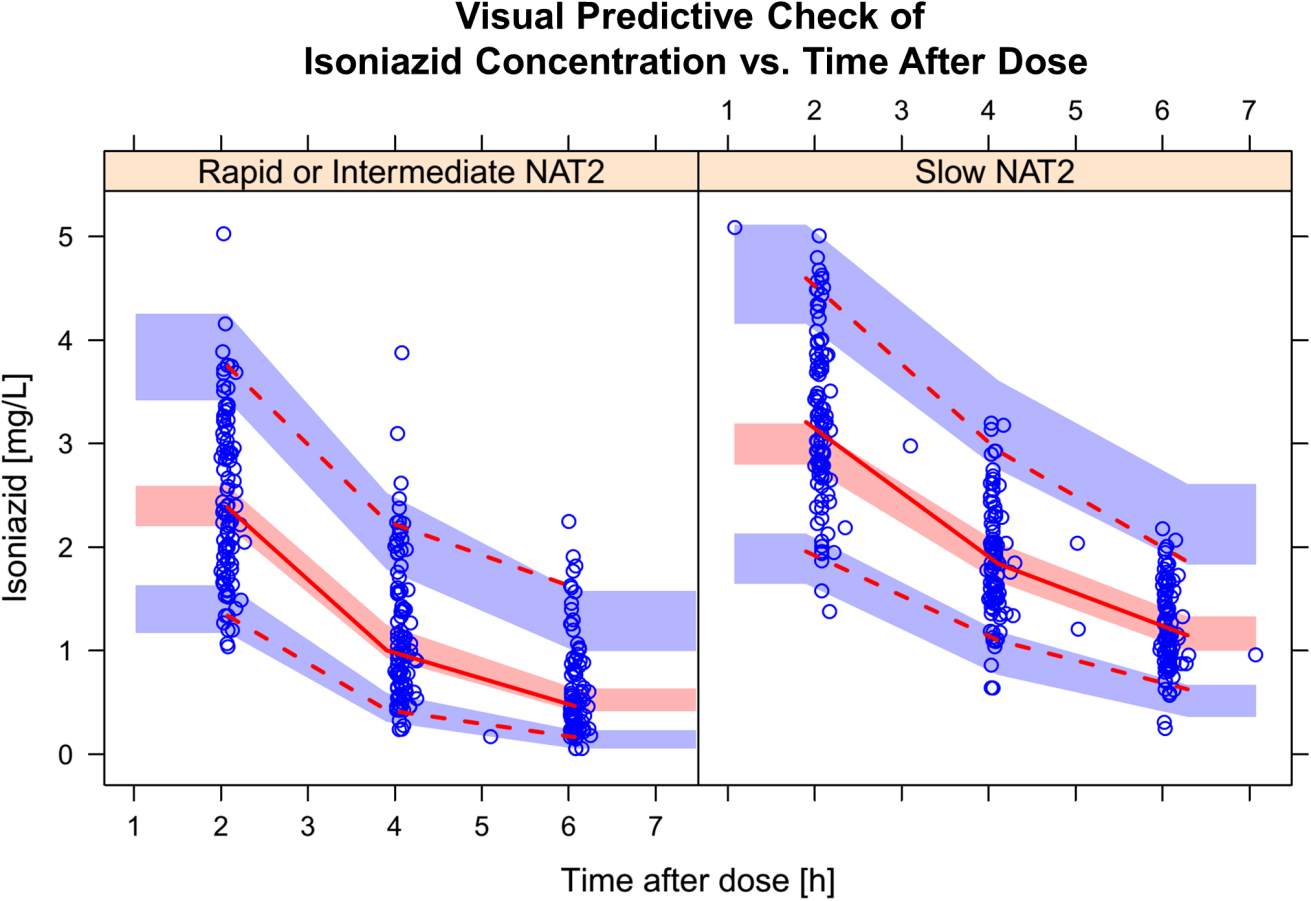
Visual predictive check (VPC) for isoniazid concentration versus time, stratified by NAT2 acetylator status (extensive and intermediate on the left, slow on the right). The circles represent the original data, the dashed and solid lines are the 5^th^, 50^th^, and 95^th^ percentiles of the original data, while the shaded areas are the corresponding 95% confidence intervals for the same percentiles, as predicted by the model.

**Fig 3:**
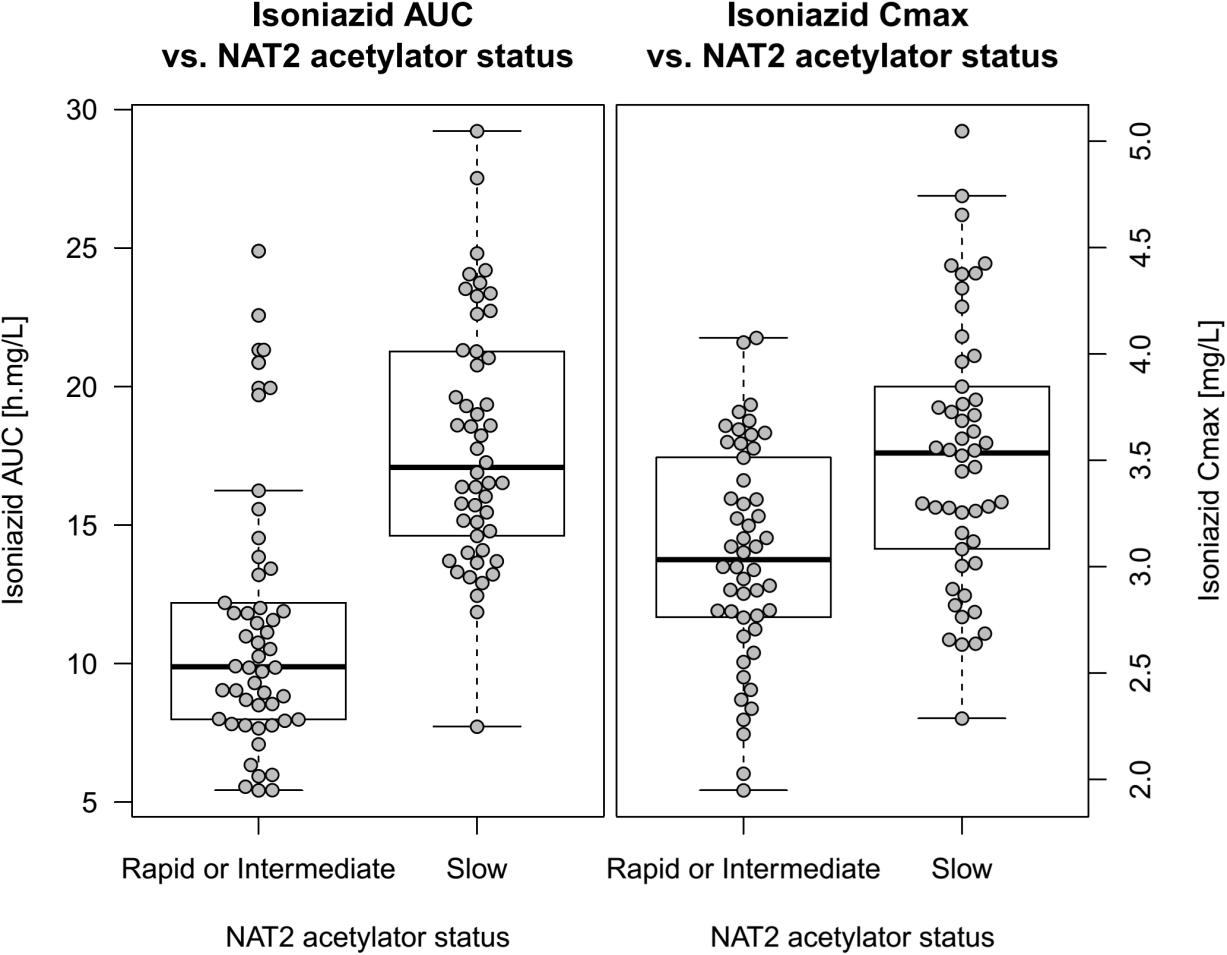
Box and whisker plots showing isoniazid exposure vs. time NAT2 acetylator status (grouped as rapid or intermediate together vs. slow). The left panel displays AUC_0-24_ and the right panel C_max_. The dots represent individual values. Since for most subjects 2 PK profiles were available, geometric mean was used to summarize the individual values.

A total of 192 isoniazid PK profiles were obtained from 100 patients, based on 574 plasma concentration measurements, three of which were BLQ. The final structural model was a two-compartment disposition with transit compartment absorption. The data did not support significantly different estimates for the absorption rate constant (k_a_) and for the rate constants between the transit compartments (k_tr_), so the absorption model was simplified. Fat-free mass (FFM) was found to be the most suitable size descriptor for allometric scaling. The model supported between-subject variability in clearance and between-occasion variability in bioavailability and mean absorption transit time (MTT). The population pharmacokinetic final parameter estimates are shown in **Table 3**, and a visual predictive check is shown in **Fig 2**.The model detected a significant effect of NAT2 acetylator status on CL (51.4 points improvement in OFV, p<10^−6^). Subjects with slow NAT2 genotype had a lower clearance (15.5 L/h) compared to rapid or intermediate NAT2 (26.1 L/h). For the two subjects with undetermined NAT2 acetylator status, their values were imputed using a mixture model taking into account both their observed isoniazid concentrations and the relative frequency of each genotype in the rest of the study population, as suggested in Keizer *et al*. [27]. The model did not detect significant effects of HIV or nutritional supplementation on clearance or bioavailability. The individual values of C_max_ and AUC_0-24h_, stratified by NAT2 genotype are shown in **Fig 3**. Among the slow NAT2 acetylators, median AUC_0-24_ and C_max_ were 17.1 h·mg/L (IQR: 14.7; 21.2) and 3.53 mg/L (IQR: 3.09; 3.83), respectively. The subjects categorised as rapid or intermediate NAT2 acetylators achieved lower median AUC_0-24_ and C_max_: 9.89 h·mg/L (IQR: 7.99; 12.1) and 3.03 mg/L (IQR: 2.76; 3.49), respectively.

**Table 3:** Isoniazid pharmacokinetics parameter estimates among newly diagnosed sputum smear positive TB patients.

Parameter description	Typical value	
	Estimate	(90% CI)^c^
Clearance for rapid/intermediate NAT2 acetylators^a^(L/h)	26.1	(23.6; 29.5)
Clearance for slow NAT2 acetylators^a^ (L/h)	15.5	(14.3; 16.7)
Central volume of distribution^a^ - Vc (L)	48.2	(18.7; 56.4)
Inter-compartmental clearance^a^ Q (L/h)	16.1	(7.5; 61.6)
Peripheral volume^a^ V_p_(L)	16.5	(12.4; 45.4)
Mean transit time “MTT” (h)	0.924	(0.78; 1.33)
Number of transit compartment–“NN”	2.73	(1.15; 5.49)
Bioavailability–“F”	1 FIXED	
Proportional error (%)	13.3	(11.7; 14.4)
Additive error (mg/L)	0.0224 FIXED	
Between subject variability of clearance^b^ (%CV)	30.7 [6%]	(24.8; 35.5)
Between occasion variability of mean transit time^b^ (%CV)	37.4 [29%]	(25.8; 41.7)
Between occasion variability of bioavailability^b^ (%CV)	12.8 [79%]	(11.1; 15.7)

^a^Allometric scaling was used for CL, Vc, Q, and Vp,so the typical values are reported for the median fat-free mass of the cohort (43 kg)

^b^The between-subject and–occasion variability was assumed log-normally distributed and is reported here as approximate %CV. In square brackets, the value of shrinkage.

^c^The precision of the estimates was obtained with a non-parametric 90% confidence interval based on a 500 sample bootstrap

For pyrazinamide and ethambutol, only116 PK profiles from 98 patients based on 346 plasma concentrations, were included in the PK modelling. Since many patients had already been switched to the continuation phase of treatment, comprised only of isoniazid and rifampicin, at the time of the second PK visit, the number of analysed patients is lower than for the isoniazid studies (n = 18). No plasma concentrations were BLQ.

**Fig 4:**
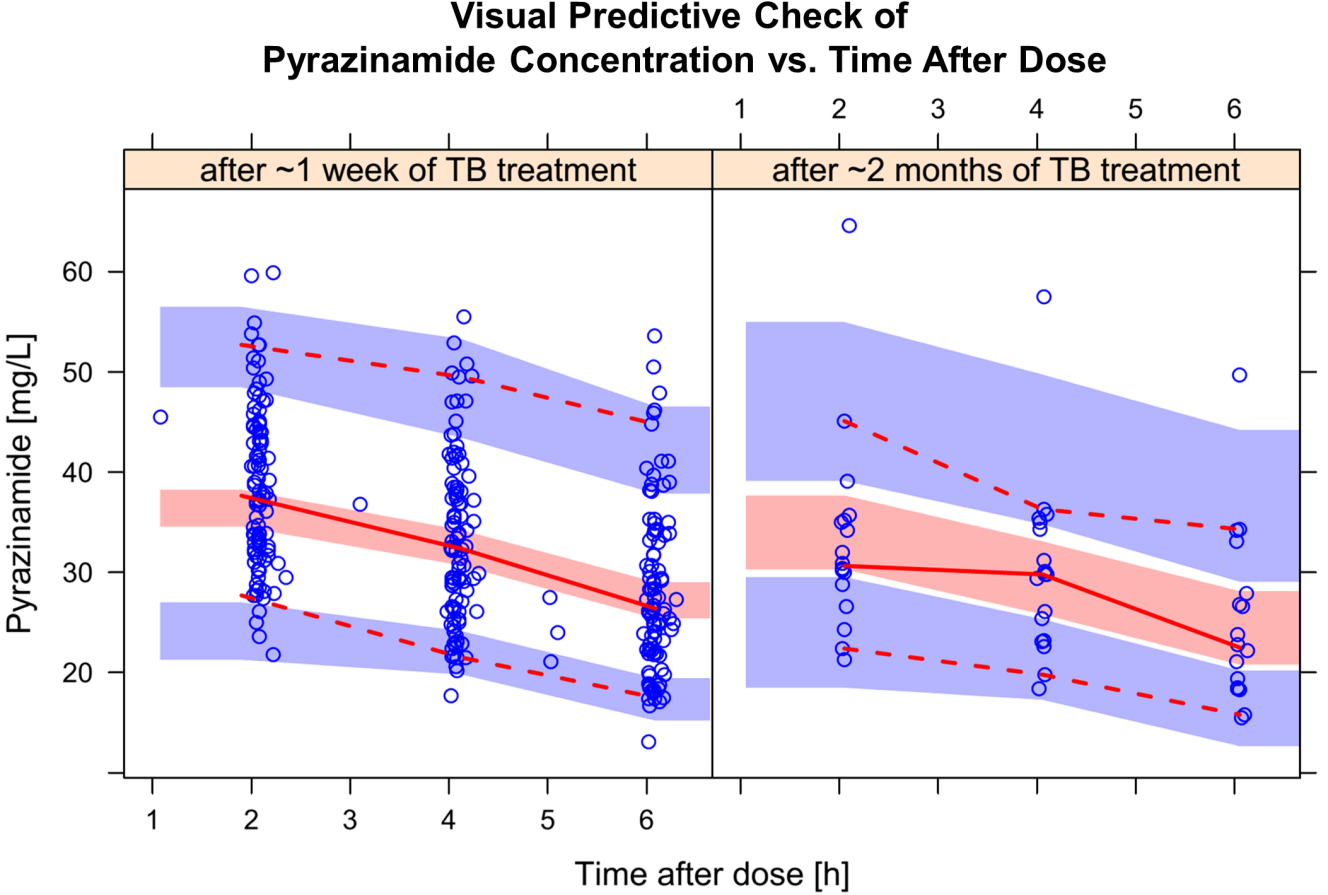
Visual predictive check (VPC) for pyrazinamide concentration versus time, stratified by time on TB treatment. The circles represent the original data, the dashed and solid lines are the 5^th^, 50^th^, and 95^th^ percentiles of the original data, while the shaded areas are the corresponding 95% confidence intervals for the same percentiles, as predicted by the model.

**Fig 5:**
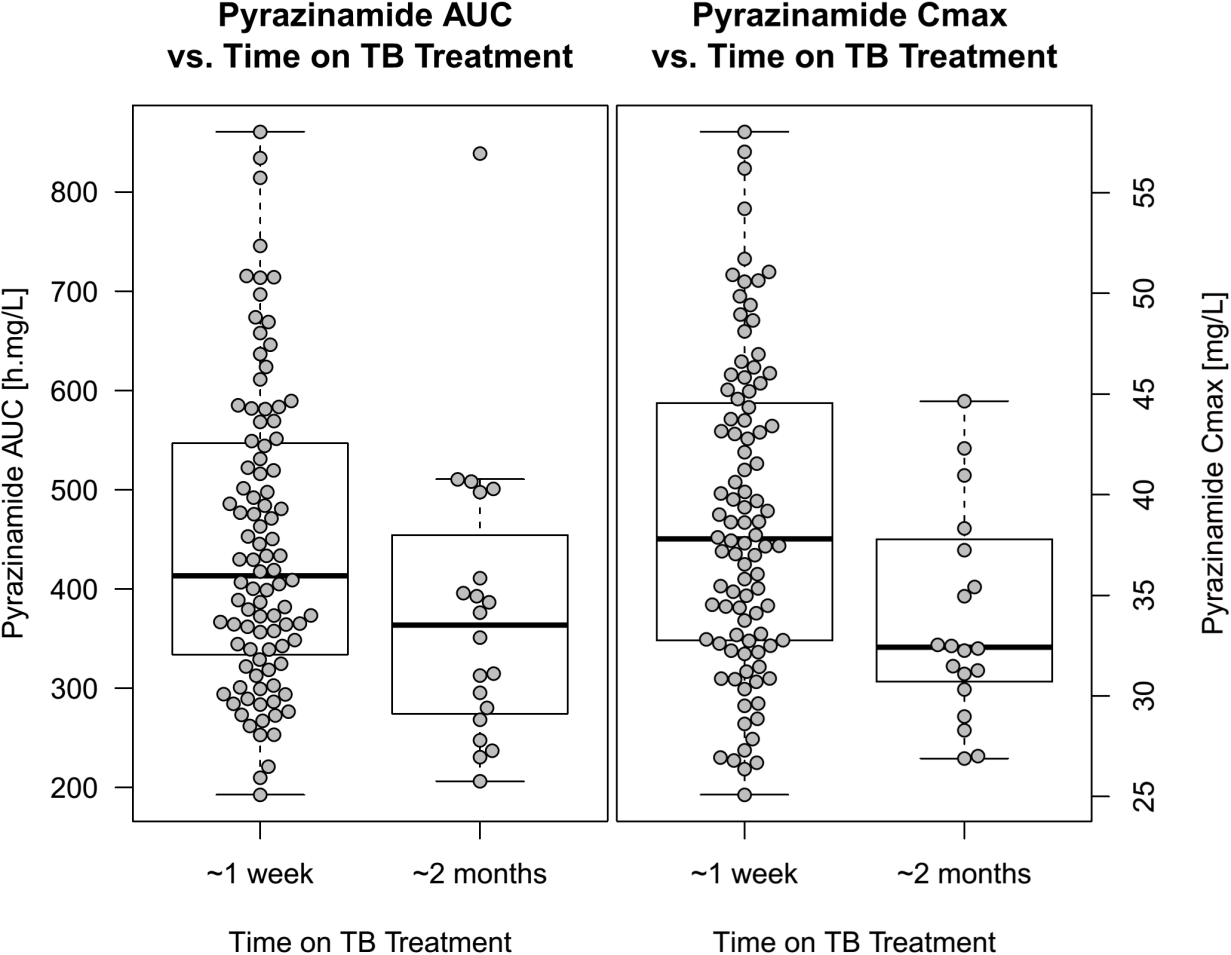
Box and whisker plots showing pyrazinamide exposure vs. time on TB treatment (approximately less or more than 2 weeks). The left panel displays AUC_0-24_ and the right panel C_max_. The dots represent individual values. When 2 PK profiles were available in the same stratum, geometric mean was used to summarize the value.

For pyrazinamide, the best model was a one-compartment disposition, first-order elimination, and transit compartment absorption with no separate estimate of absorption rate constants (k_transit_ = k_a_). The final parameter estimates are included in **Table 4,** while a visual predictive check is shown in **Fig 4**. The best size predictor for allometric scaling of clearance was total body weight, while volume of distribution was better scaled with fat-free mass. Pyrazinamide clearance increased with time on treatment: the model estimated 16.3% faster clearance from the data collected after more than 18 days of treatment (-6.96 OFV, p<0.01). This break point was chosen to include all PK profiles from the second PK visit, mostly collected 2 months after treatment initiation, plus two late-comers for the first PK occasion (on days 19 and 26). Other factors including HIV status, nutritional supplementation, age, sex, CD4 count, and weight-adjusted dose were tested in the model, but did not significantly influence pyrazinamide PK. The relationship between these individual values of pyrazinamide exposure and time on TB treatment is shown in **Fig 5**. Among the PK profiles obtained in the first 2 weeks of TB treatment, median pyrazinamide AUC_0-24_ and C_max_ were 413 h·mg/L (IQR: 337; 546) and 37.8 mg/L (IQR: 32.8; 44.5), respectively. For the profiles collected after 2 weeks of TB treatment AUC_0-24_ and C_max_ decreased to 364 h·mg/L (IQR: 277; 433) and 32.4 mg/L (IQR: 30.9; 37.5), respectively.

**Table 4:** Pyrazinamide pharmacokinetics parameter estimates among newly diagnosed sputum smear positive pulmonary TB patients.

Parameter description	Typical value	
	Estimate	(90% CI)^d^
Clearance^a^–CL (L/h)	3.32	(3.10; 3.53)
Volume of distribution^a^ –V_d_ (L)	40.1	(38.4; 42.1)
Absorption mean transit time—“MTT” (h)	0.84	(0.42; 1.08)
Number of absorption transit compartments–NN	2.6	(0.2; 7.3)
Bioavailability–F	1 FIXED	
Clearance change after 2 months of treatment^b^ (+%)	16.3	(2.6; 29.2)
Proportional error (%)	7.2	(6.0; 8.1)
Additive error (mg/L)	0.041 FIXED	
Between-subject variability in clearance^c^ (%CV)	22.6 [28%]	(11.7; 30.8)
Between-occasion variability in clearance^c^ (%CV)	19.3 [40%]	(2.0; 28.2)
Between-occasion variability in mean transit time^c^ (%CV)	46.9 [38%]	(32.3; 89.9)
Between-occasion variability in bioavailability^c^ (%CV)	10.1 [39%]	(4.6; 13.2)

^a^Allometric scaling was used for clearance (total weight) and volume of distribution (fat-free mass), so the values are reported for the median weight (52 kg) and fat-free mass (43 kg) of the cohort.

^b^Although nearly all the profiles with increased clearance were collected at ~2 months after TB treatment initiation, the cut-off used in the model was 18 days.

^c^The between-subject and–occasion variability was assumed log-normally distributed and is reported here as approximate %CV. In square brackets, the value of shrinkage.

^d^The precision of the estimates was obtained with a non-parametric 90% confidence interval based on a 500-sample bootstrap.

**Fig 6:**
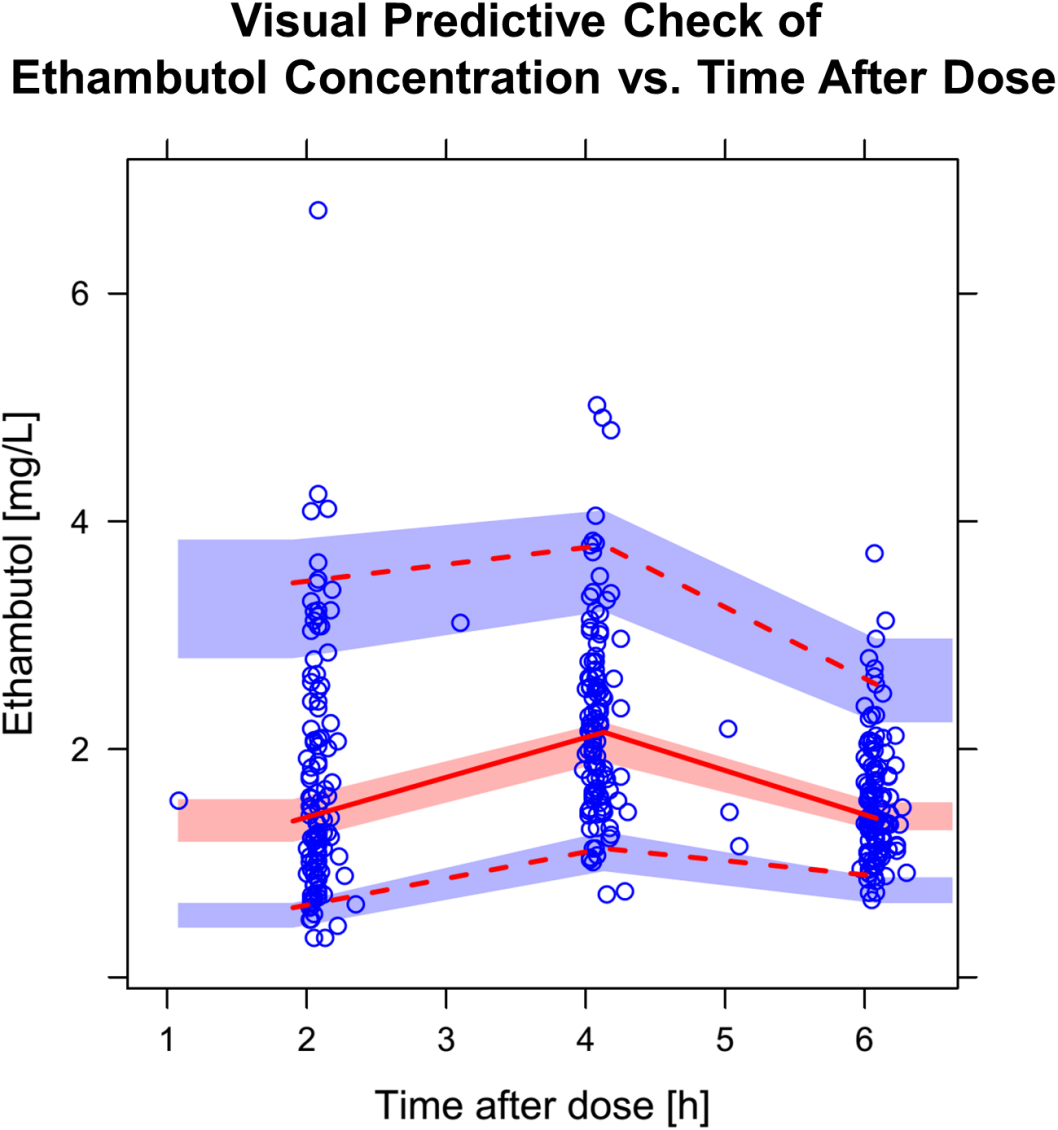
Visual predictive check (VPC) for ethambutol concentration versus time. The circles represent the original data, the dashed and solid lines are the 5^th^, 50^th^, and 95^th^ percentiles of the original data, while the shaded areas are the corresponding 95% confidence intervals for the same percentiles, as predicted by the model.

**Fig 7:**
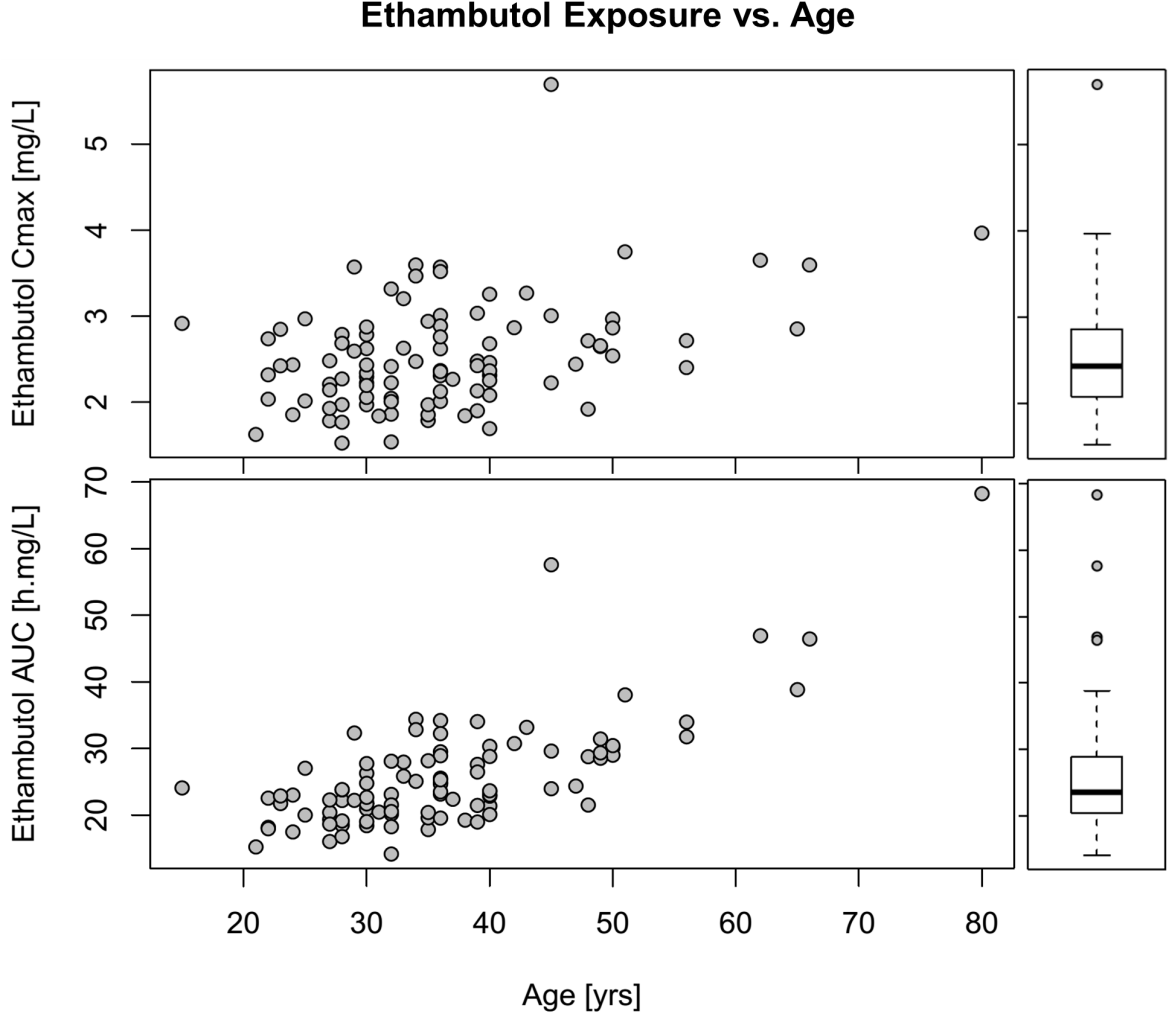
In the left panels, scatter plots showing ethambutol exposure vs. patient age. In the right small panels, box and whiskers plots summarizing the same values. The top panels refer to AUC_0-24_ and the bottom panels to C_max_. For all patients for whom 2 PK profiles were available, geometric mean was used to obtain summary values.

For ethambutol, the best-fitting model was a two-compartment disposition, with first-order elimination, and transit compartment absorption with no separate estimate of k_a_. The final parameter estimates are shown in **Table 5**and a visual predictive check is shown in **Fig 6**. Although the inclusion of two-compartment disposition kinetics significantly improved the model fit (-30 points OFV), the parameter estimates for the volume of the peripheral compartment (Vp) and the inter-compartmental clearance (Q) proved unstable. To stabilise the model while allowing the inclusion of the two-compartment kinetics, a prior was included [28], based on parameter estimates from a PK model of ethambutol developed on data from a similar population of TB patients [29]. After applying allometric scaling to adjust for differences in body weight amongst the studies, the typical values for the priors of Vp and Q were 420.7 L/h and 64.4 L/h, respectively. The priors were assumed to have a Gaussian distribution around these typical values, and were included in the model imputing a large uncertainty (50% CV) to make them weakly informative. Testing different settings for the prior distributions showed that the estimates of the other parameters in the model were not significantly affected. After the inclusion of the priors, the two-compartment model proved stable and provided a significantly better fit than the one-compartment model, and was used for the analysis. The best predictor for allometric scaling of all clearance and volume parameters was total body weight. Additionally, older age was associated with lower clearance, with every year of age causing a decrease of 1.41% in clearance (-23.4 OFV, p<10 ^-5^). No other factors tested in the model, including HIV status, nutritional supplementation, sex, CD4 count, and time on TB treatment, significantly affected the PK. The relationship between the individual values of ethambutol exposure and age is shown in **Fig 7**. Median ethambutol AUC_0-24_ was 23.6 h·mg/L (IQR: 20.5; 28.9) and C_max_ was 2.44 mg/L (IQR: 2.09; 2.86).

**Table 5:** Ethambutol pharmacokinetics parameter estimates among newly diagnosed sputum smear positive pulmonary TB patients.

Parameter description	Typical value	
	Estimate	(90% CI)^e^
Clearance^a^ ^,^ ^b^ –CL (L/h)	40.7	(35.7; 45.2)
Central volume of distribution^a^ - Vc (L)	266	(207; 326)
Inter-compartmental clearance^a^ ^,^ ^c^ - Q (L/h)	109	(82; 136)
Peripheral volume of distribution^a^ ^,^ ^c^ - Vp (L)	687	(493; 850)
Absorption mean transit time—“MTT” (h)	2.54	(2.32; 2.78)
Number of absorption transit compartments–NN	11.1	(6.0; 30.2)
Bioavailability–F	1 FIXED	
Effect of age on Clearance (% change per year)	-1.41	(-1.76; -1.09)
Proportional error (%)	22.5	(19.1; 24.5)
Additive error (mg/L)	0.0162 FIXED	
Between-subject variability in bioavailability^d^ (%CV)	21.5 [14%]	(15.7; 26.2)
Between-occasion variability in mean transit time^d^ (%CV)	26.1 [14%]	(16.9; 33.7)

^a^Allometric scaling was used for CL, Vc, Q, and Vp, so the values are reported for the median weight of the cohort (52 kg).

^b^CL was affected by age, the typical value reported here refers to the median age in the cohort (35 years)

^c^Q and Vp were estimated using Gaussian priors with typical values 64.4 L/h and 420.7 L respectively, and 50% uncertainty.

^d^The between-subject and–occasion variability was assumed log-normally distributed and is reported here as approximate %CV. In square brackets, the value of shrinkage.

^e^The precision of the estimates was obtained with a non-parametric 90% confidence interval based on a 500 sample bootstrap

## Discussion

We studied the effect of nutritional supplementation and HIV status on the pharmacokinetics of isoniazid, pyrazinamide, and ethambutol in pulmonary TB patients during the intensive phase of a standard course of TB treatment.

Malnutrition is a well-known companion to both HIV and TB, and food programs are therefore being launched in many Sub-Saharan regions to alleviate this problem. Nutritional supplementation has previously been reported to improve treatment outcome in both TB and HIV patients [30, 31], so our study aimed to investigate if nutritional intervention is affecting the PK exposure of the first-line TB drugs. We recently published the beneficial effect of nutritional supplementation on rifampicin exposure, especially in HIV positive TB patients [20]. In the current study, we found that nutritional supplementation had no effect on isoniazid, pyrazinamide, and ethambutol exposure, and there was no effect of HIV co-infection. The effect of nutritional supplements may depend on the individual’s baseline micronutrients status and only cause an effect in undernourished subjects [32]. In our cohort, baseline BMI was 18.8 (IQR 17.3; 19.9) which classifies most of the participants in the category of underweight; however, we did not assess their micronutrient status.

We further assessed predictors that potentially could influence the PK, including the NAT2 genotype, age of the patient, and timing of the sampling with respect to treatment initiation. The NAT2 gene product is expressed in the liver and small intestine, constituting an important phase II enzyme responsible for acetylating isoniazid [33]. NAT2 activities may vary due to differences in the NAT2 alleles or haplotypes caused by Single-Nucleotide Polymorphisms (SNPs) [33]. As expected, NAT2 genotype strongly influenced isoniazid pharmacokinetics. Patients categorized as slow NAT2 acetylators had a lower clearance (typical value 15.5 vs. 26.1 L/h) and higher estimated isoniazid exposures (AUC_0-24_ of 17.1 vs. 9.89 h·mg/L).compared to rapid or intermediate NAT2 acetylators. The distribution of NAT2 genotypes is comparable to what was found by Sabbagh *et al*. and Matimba *et al*. who reported a high prevalence of slow and intermediate acetylators in African populations, due to the common NAT2*5, *6, and *14 polymorphisms [34, 35]. Our findings are in line with those reported by Conte *et al*. examining the effects of gender, AIDS, and acetylator status on the steady-state concentrations of orally administered isoniazid in plasma and lungs [36]. Similar findings were reported by Chen *et al*. assessing the influence of NAT2 genotype on the plasma concentration of isoniazid and acetyl-isoniazid in a Chinese population [37]. Pasipanodya *et al*. have compiled these results in a meta-analysis and suggest that this genetic variability is a contributing factor for microbiological treatment failure [9].

When characterising pyrazinamide PK, we found that clearance increases with time on treatment, an observation also recently reported by Chirehwa *et al*, in South African TB patients co-infected with HIV [38]. They detected an increase of 19% in clearance by day 28 after treatment initiation, which is comparable with our finding of a 16.3% increase. During TB treatment, pyrazinamide is given concomitantly (or even co-formulated) with rifampicin, which is a well-known potent inducer of hepatic and intestinal CYP3A subfamily and many other metabolic pathways via activation of the pregnane X-receptor (PXR) [39]. For this reason, rifampicin exposure results in increasing clearance of many co-administered drugs, and it could be speculated that rifampicin may induce microsomal deamidase or some other pathway, thus enhancing pyrazinamide clearance [38]. On the other hand, the observed increase in clearance could also be the effect of the overall improvement in health conditions of the patients after treatment initiation. The pyrazinamide exposures we observed did not significantly deviate from previous results [40–44]. In the current study, the estimates for median pyrazinamide C_max_ and AUC_0-24_ were in line with previous reports showing median C_max_ levels ranging from 27 to 38 mg/L and AUC_0-24_ between 321 and 418 h·mg/L [41–44]. Our results also confirm the reports by Fahimi *et al*. and Tappero *et al*. who showed that the majority of patients achieve pyrazinamide plasma exposures within a range relatively narrower than other TB drugs, due to its efficient absorption [45, 46]. Pasipanodya *et al*. recently investigated the TB drug concentration levels that are predictive of TB treatment outcome, and they reported that pyrazinamide peak concentration ≤ 58.3 mg/L was associated with poor 2-month sputum conversion, while AUC_0-24_ ≤ 363 h·mg/L was one of the predictors of poor long term outcome [7]. In their analysis, PK exposures were measured at 2 months after treatment initiation. In our cohort, only one PK profile had C_max_ above the proposed threshold at around 2 months after treatment initiation. The overall median C_max_ was 32.4 mg/L, while the median pyrazinamide AUC_0-24_ at around 2 months after treatment initiation was 364 h·mg/L, and a similar value is obtained when adjusting the median AUC_0-24_ observed after 1 week to account for the estimated increase in clearance, i.e., multiplying by 1/(1+16.3%). More specifically, we found that 31.6% of this population had pyrazinamide AUC_0-24_ ≤ 363 h·mg/L around 1 week and 55.6% at around 2 months after TB treatment initiation. This means that about half of the patients in our cohort achieved exposures below the proposed AUC threshold, and nearly none achieved C_max_ above the cut-off. Unfortunately, our study was not powered to assess the long-term effect of drug exposure on treatment outcome.

Ethambutol plasma concentrations among our study participants were relatively low compared with previous studies [10, 40–42, 44], reporting median C_max_ ranging from 2.7 to 4.8 mg/L and median AUC_0-24_ between 20 and 47 h·mg/L. In our cohort, median ethambutol C_max_ was 2.44 mg/L and AUC_0-24_ was 23.6 h·mg/L, values similar to those reported by Tappero *et al*. and Um *et al*. [46, 47]. Ethambutol pharmacokinetics has been previously associated with many factors including malnutrition, HIV infection, age, and sex [12, 42, 48–50]. In our cohort, age was found to affect ethambutol clearance, with increasing age leading to lower clearance at a decrease rate of 1.41% per year. A relationship between age and anti-TB drug plasma levels has been previously reported in a study in South African patients [12], where it was suggested that older patients have higher levels of ethambutol and isoniazid because of the functional decrease of metabolic pathways and reduced renal clearance capacity. HIV infection was not found to affect ethambutol plasma concentration in this population, in contrast to studies by Zhu *et al*. [51] and Jonsson *et al*. [10], who both reported that HIV infection was associated with a reduction in ethambutol concentrations.

### Limitations and strengths

The number of PK samples collected at each visit was small, limiting the characterization of the pharmacokinetic curve, as well as the precision of the individual estimates of exposure, especially C_max_. This was a compromise accepted in the study design to limit the time patients had to spend at the clinic during PK sampling, as the patients involved were treated as outpatients. The data was interpreted with nonlinear mixed-effects modelling, which appropriately handles sparse sampling and supports the robustness of our findings.

Another limitation of the study is represented by the few PK profiles of pyrazinamide and ethambutol available from the second visit (only 18 out of those that came back for the second evaluation), since a majority of the patients had already been switched to the continuation phase not including pyrazinamide and ethambutol. This missingness of the data reduced the sample size for the investigation of the effect of the time on treatment, but nonlinear mixed-effects modelling is known to handle these kinds of scenarios well. Moreover, the two cohorts (patients in continuation vs intensive phase at PK visit 2) had similar demographic characteristics and similar proportions of HIV infection and subjects randomised to supplementation (data not shown).

This study was initiated and conducted before clear policies regarding the timing of ART to HIV co-infected TB patients were established. Therefore pharmacologic interaction with various ARTs is not an issue in this study.

## Conclusions

In summary, we reported the pharmacokinetics of isoniazid, pyrazinamide, and ethambutol in a cohort of Tanzanian TB patients. We found that nutritional supplements with energy-protein plus micronutrients given to TB patients during the intensive phase of a conventional TB regimen have no effect on the exposure of isoniazid, pyrazinamide, or ethambutol. HIV status did not influence this result. Intermediate and rapid NAT2 genotypes were associated with lower isoniazid exposure. Pyrazinamide clearance increased with time on treatment, which was associated with lower serum pyrazinamide levels at the end of intensive phase of TB treatment. Ethambutol plasma concentrations were relatively low in our cohort of Tanzanian patients compared with previous studies, and older age was associated with lower clearance of ethambutol.

## Supporting Information

S1 Protocol Supplementary PDF file with the study protocol. (DOC)

## References

[1] Wada M, Yoshiyama T, Ogata H, Ito K, Mizutani S, Sugita H. [Six-months chemotherapy (2HRZS or E/4HRE) of new cases of pulmonary tuberculosis—six year experiences on its effectiveness, toxicity, and acceptability]. Kekkaku. 1999;74(4):353–60. Epub 1999/06/04. . 10355221

[2] Treatment of tuberculosis and tuberculosis infection in adults and children. American Thoracic Society. Monaldi Arch Chest Dis. 49(4):327–45. Epub 1994/09/01. . 8000420

[3] Fattorini L, Piccaro G, Mustazzolu A, Giannoni F. Targeting Dormant Bacilli to Fight Tuberculosis. Mediterr J Hematol Infect Dis. 2013;5(1):e2013072 Epub 2013/12/24. 10.4084/mjhid.2013.072 24363887 PMC3867226

[4] World Health Organization. Global tuberculosis report 2013 Geneva, Switzerland: World Health Organization; 2013.

[5] Ministry of Health and Social welfare. Manual of the national tuberculosis and leprosy programme in Tanzania Dar es Salaam: 2013 [cited 2014 30 November]. Sixth:[Available from: ntlp.go.tz/index.php?option=com_phocadownload&view…

[6] Peloquin CA. Therapeutic drug monitoring in the treatment of tuberculosis. Drugs. 2002;62(15):2169–83. Epub 2002/10/17. . 12381217 10.2165/00003495-200262150-00001

[7] Pasipanodya JG, McIlleron H, Burger A, Wash PA, Smith P, Gumbo T. Serum drug concentrations predictive of pulmonary tuberculosis outcomes. J Infect Dis. 2013;208(9):1464–73. Epub 2013/08/01. 10.1093/infdis/jit352 23901086 PMC3789573

[8] Srivastava S, Pasipanodya JG, Meek C, Leff R, Gumbo T. Multidrug-resistant tuberculosis not due to noncompliance but to between-patient pharmacokinetic variability. J Infect Dis. 2011;204(12):1951–9. Epub 2011/10/25. 10.1093/infdis/jir658 22021624 PMC3209814

[9] Pasipanodya JG, Srivastava S, Gumbo T. Meta-analysis of clinical studies supports the pharmacokinetic variability hypothesis for acquired drug resistance and failure of antituberculosis therapy. Clin Infect Dis. 2012;55(2):169–77. Epub 2012/04/03. 10.1093/cid/cis353 22467670 PMC3491771

[10] Jonsson S, Davidse A, Wilkins J, Van der Walt JS, Simonsson US, Karlsson MO, et al Population pharmacokinetics of ethambutol in South African tuberculosis patients. Antimicrob Agents Chemother. 2011;55(9):4230–7. Epub 2011/06/22. 10.1128/aac.00274-11 21690284 PMC3165318

[11] Wilkins JJ, Langdon G, McIlleron H, Pillai GC, Smith PJ, Simonsson US. Variability in the population pharmacokinetics of pyrazinamide in South African tuberculosis patients. Eur J Clin Pharmacol. 2006;62(9):727–35. Epub 2006/05/11. 10.1007/s00228-006-0141-z . 16685561

[12] McIlleron H, Wash P, Burger A, Norman J, Folb PI, Smith P. Determinants of rifampin, isoniazid, pyrazinamide, and ethambutol pharmacokinetics in a cohort of tuberculosis patients. Antimicrob Agents Chemother. 2006;50(4):1170–7. Epub 2006/03/30. 10.1128/aac.50.4.1170-1177.2006 16569826 PMC1426981

[13] Chideya S, Winston CA, Peloquin CA, Bradford WZ, Hopewell PC, Wells CD, et al Isoniazid, rifampin, ethambutol, and pyrazinamide pharmacokinetics and treatment outcomes among a predominantly HIV-infected cohort of adults with tuberculosis from Botswana. Clin Infect Dis. 2009;48(12):1685–94. Epub 2009/05/13. 10.1086/599040 19432554 PMC3762461

[14] Graham SM, Bell DJ, Nyirongo S, Hartkoorn R, Ward SA, Molyneux EM. Low levels of pyrazinamide and ethambutol in children with tuberculosis and impact of age, nutritional status, and human immunodeficiency virus infection. Antimicrob Agents Chemother. 2006;50(2):407–13. Epub 2006/01/27. 10.1128/aac.50.2.407-413.2006 16436690 PMC1366879

[15] Perlman DC, Segal Y, Rosenkranz S, Rainey PM, Remmel RP, Salomon N, et al The clinical pharmacokinetics of rifampin and ethambutol in HIV-infected persons with tuberculosis. Clin Infect Dis. 2005;41(11):1638–47. Epub 2005/11/04. 10.1086/498024 . 16267738

[16] Gurumurthy P, Ramachandran G, Hemanth Kumar AK, Rajasekaran S, Padmapriyadarsini C, Swaminathan S, et al Malabsorption of rifampin and isoniazid in HIV-infected patients with and without tuberculosis. Clin Infect Dis. 2004;38(2):280–3. Epub 2003/12/31. 10.1086/380795 . 14699462

[17] Singh N, Dubey S, Chinnaraj S, Golani A, Maitra A. Study of NAT2 gene polymorphisms in an Indian population: association with plasma isoniazid concentration in a cohort of tuberculosis patients. Mol Diagn Ther. 2009;13(1):49–58. Epub 2009/04/09. 10.2165/01250444-200913010-00007 . 19351215

[18] Oshikoya KA, Sammons HM, Choonara I. A systematic review of pharmacokinetics studies in children with protein-energy malnutrition. Eur J Clin Pharmacol. 2010;66(10):1025–35. Epub 2010/06/17. 10.1007/s00228-010-0851-0 . 20552179

[19] Buchanan N, Eyberg C, Davis MD. Isoniazid pharmacokinetics in kwashiorkor. S Afr Med J. 1979;56(8):299–300. Epub 1979/08/25. . 550491

[20] Jeremiah K, Denti P, Chigutsa E, Faurholt-Jepsen D, PrayGod G, Range N, et al Nutritional supplementation increases rifampin exposure among tuberculosis patients coinfected with HIV. Antimicrob Agents Chemother. 2014;58(6):3468–74. Epub 2014/04/09. 10.1128/aac.02307-13 24709267 PMC4068463

[21] NACP. National AIDS Control Programme (NACP) National Guidelines For the Management of HIV and AIDS, Government of Tanzania 2009.

[22] Beal S, Sheiner L., Boeckmann A., & Bauer R. (NONMEM users guides (1989–2009). Ellicott City, MD, USA,: ICON Development Solution; 2009.

[23] Keizer RJ, Karlsson MO, Hooker A. Modeling and Simulation Workbench for NONMEM: Tutorial on Pirana, PsN, and Xpose. CPT: pharmacometrics & systems pharmacology. 2013;2:e50 Epub 2013/07/10. 10.1038/psp.2013.24 23836189 PMC3697037

[24] Savic RM, Jonker DM, Kerbusch T, Karlsson MO. Implementation of a transit compartment model for describing drug absorption in pharmacokinetic studies. J Pharmacokinet Pharmacodyn. 2007;34(5):711–26. Epub 2007/07/27. 10.1007/s10928-007-9066-0 . 17653836

[25] Anderson BJ, Holford NH. Mechanism-based concepts of size and maturity in pharmacokinetics. Annu Rev Pharmacol Toxicol. 2008;48:303–32. Epub 2007/10/05. 10.1146/annurev.pharmtox.48.113006.094708 . 17914927

[26] Savic RM, Karlsson MO. Importance of shrinkage in empirical bayes estimates for diagnostics: problems and solutions. The AAPS journal. 2009;11(3):558–69. Epub 2009/08/04. 10.1208/s12248-009-9133-0 19649712 PMC2758126

[27] Keizer RJ, Zandvliet AS, Beijnen JH, Schellens JH, Huitema AD. Performance of methods for handling missing categorical covariate data in population pharmacokinetic analyses. The AAPS journal. 2012;14(3):601–11. Epub 2012/06/01. 10.1208/s12248-012-9373-2 22648902 PMC3385822

[28] Gisleskog PO, Karlsson MO, Beal SL. Use of prior information to stabilize a population data analysis. J Pharmacokinet Pharmacodyn. 2002;29(5–6):473–505. Epub 2003/06/11. . 12795242 10.1023/a:1022972420004

[29] Merle CS, Fielding K, Sow OB, Gninafon M, Lo MB, Mthiyane T, et al A four-month gatifloxacin-containing regimen for treating tuberculosis. N Engl J Med. 2014;371(17):1588–98. Epub 2014/10/23. 10.1056/NEJMoa1315817 . 25337748

[30] Karyadi E, West CE, Schultink W, Nelwan RH, Gross R, Amin Z, et al A double-blind, placebo-controlled study of vitamin A and zinc supplementation in persons with tuberculosis in Indonesia: effects on clinical response and nutritional status. Am J Clin Nutr. 2002;75(4):720–7. Epub 2002/03/28. . 11916759 10.1093/ajcn/75.4.720

[31] Ramakrishnan CV, Rajendran K, Jacob PG, Fox W, Radhakrishna S. The role of diet in the treatment of pulmonary tuberculosis. An evaluation in a controlled chemotherapy study in home and sanatorium patients in South India. Bull World Health Organ. 1961;25:339–59. Epub 1961/01/01. 14490066 PMC2555577

[32] Friis H. Micronutrient interventions and HIV infection: a review of current evidence. Trop Med Int Health. 2006;11(12):1849–57. Epub 2006/12/21. 10.1111/j.1365-3156.2006.01740.x . 17176350

[33] Tilak AV, Iyer SN, Mukherjee MS, Singhal RS, Lele SS. Full-gene-sequencing analysis of N-acetyltransferase-2 in an adult Indian population. Genetic testing and molecular biomarkers. 2013;17(3):188–94. Epub 2012/12/12. 10.1089/gtmb.2012.0258 . 23216273

[34] Matimba A, Del-Favero J, Van Broeckhoven C, Masimirembwa C. Novel variants of major drug-metabolising enzyme genes in diverse African populations and their predicted functional effects. Human genomics. 2009;3(2):169–90. Epub 2009/01/24. 19164093 10.1186/1479-7364-3-2-169 PMC3525272

[35] Sabbagh A, Langaney A, Darlu P, Gerard N, Krishnamoorthy R, Poloni ES. Worldwide distribution of NAT2 diversity: implications for NAT2 evolutionary history. BMC Genet. 2008;9:21 Epub 2008/02/29. 10.1186/1471-2156-9-21 18304320 PMC2292740

[36] Conte JE Jr., Golden JA, McQuitty M, Kipps J, Duncan S, McKenna E, et al Effects of gender, AIDS, and acetylator status on intrapulmonary concentrations of isoniazid. Antimicrob Agents Chemother. 2002;46(8):2358–64. Epub 2002/07/18. 12121905 10.1128/AAC.46.8.2358-2364.2002 PMC127347

[37] Chen B, Li JH, Xu YM, Wang J, Cao XM. The influence of NAT2 genotypes on the plasma concentration of isoniazid and acetylisoniazid in Chinese pulmonary tuberculosis patients. Clin Chim Acta. 2006;365(1–2):104–8. Epub 2005/09/27. 10.1016/j.cca.2005.08.012 . 16182272

[38] Chirehwa MR, R. Mthiyane, T. Onyebujoh, P. Smith, P. McIlleron, H. & Denti,. Population pharmacokinetics of Pyrazinamide among HIV/TB co-infected patients at different levels of immunosuppression in South Africa. In World Congress of Pharmacology. 2014:(p. Poster 265). Cape Town, South Africa.

[39] Niemi M, Backman JT, Fromm MF, Neuvonen PJ, Kivisto KT. Pharmacokinetic interactions with rifampicin: clinical relevance. Clin Pharmacokinet. 2003;42(9):819–50. Epub 2003/07/29. 10.2165/00003088-200342090-00003 . 12882588

[40] Babalik A, Ulus IH, Bakirci N, Kuyucu T, Arpag H, Dagyildiz L, et al Pharmacokinetics and serum concentrations of antimycobacterial drugs in adult Turkish patients. Int J Tuberc Lung Dis. 2013;17(11):1442–7. Epub 2013/10/16. 10.5588/ijtld.12.0771 . 24125448

[41] Chigutsa E. Population pharmacokinetics and pharmacokinetic-pharmacodyamic modeling of antitubercular drugs University of Cape Town, SA University of Cape Town; 2013.

[42] Peloquin CA, Bulpitt AE, Jaresko GS, Jelliffe RW, Childs JM, Nix DE. Pharmacokinetics of ethambutol under fasting conditions, with food, and with antacids. Antimicrob Agents Chemother. 1999;43(3):568–72. Epub 1999/02/27. 10049268 10.1128/aac.43.3.568 PMC89161

[43] Peloquin CA, Jaresko GS, Yong CL, Keung AC, Bulpitt AE, Jelliffe RW. Population pharmacokinetic modeling of isoniazid, rifampin, and pyrazinamide. Antimicrob Agents Chemother. 1997;41(12):2670–9. Epub 1998/01/07. 9420037 10.1128/aac.41.12.2670 PMC164187

[44] Tostmann A, Mtabho CM, Semvua HH, van den Boogaard J, Kibiki GS, Boeree MJ, et al Pharmacokinetics of first-line tuberculosis drugs in Tanzanian patients. Antimicrob Agents Chemother. 2013;57(7):3208–13. Epub 2013/05/01. 10.1128/aac.02599-12 23629715 PMC3697325

[45] Fahimi F, Tabarsi P, Kobarfard F, Bozorg BD, Goodarzi A, Dastan F, et al Isoniazid, rifampicin and pyrazinamide plasma concentrations 2 and 6 h post dose in patients with pulmonary tuberculosis. Int J Tuberc Lung Dis. 2013;17(12):1602–6. Epub 2013/11/10. 10.5588/ijtld.13.0019 . 24200276

[46] Tappero JW, Bradford WZ, Agerton TB, Hopewell P, Reingold AL, Lockman S, et al Serum concentrations of antimycobacterial drugs in patients with pulmonary tuberculosis in Botswana. Clin Infect Dis. 2005;41(4):461–9. Epub 2005/07/20. 10.1086/431984 . 16028152

[47] Um SW, Lee SW, Kwon SY, Yoon HI, Park KU, Song J, et al Low serum concentrations of anti-tuberculosis drugs and determinants of their serum levels. Int J Tuberc Lung Dis. 2007;11(9):972–8. Epub 2007/08/21. . 17705974

[48] Kimerling ME, Phillips P, Patterson P, Hall M, Robinson CA, Dunlap NE. Low serum antimycobacterial drug levels in non-HIV-infected tuberculosis patients. Chest. 1998;113(5):1178–83. Epub 1998/05/22. . 9596291 10.1378/chest.113.5.1178

[49] McIlleron H, Rustomjee R, Vahedi M, Mthiyane T, Denti P, Connolly C, et al Reduced antituberculosis drug concentrations in HIV-infected patients who are men or have low weight: implications for international dosing guidelines. Antimicrob Agents Chemother. 2012;56(6):3232–8. Epub 2012/03/14. 10.1128/aac.05526-11 22411614 PMC3370773

[50] Peloquin CA, Bulpitt AE, Jaresko GS, Jelliffe RW, James GT, Nix DE. Pharmacokinetics of pyrazinamide under fasting conditions, with food, and with antacids. Pharmacotherapy. 1998;18(6):1205–11. Epub 1998/12/17. . 9855317

[51] Zhu M, Burman WJ, Starke JR, Stambaugh JJ, Steiner P, Bulpitt AE, et al Pharmacokinetics of ethambutol in children and adults with tuberculosis. Int J Tuberc Lung Dis. 2004;8(11):1360–7. Epub 2004/12/08. . 15581206

