# Supplementary material for: Pharmacokinetics of Isoniazid, Pyrazinamide, and Ethambutol in Newly Diagnosed Pulmonary TB Patients in Tanzania
Source: PLoS One. 2015 Oct 26;10(10):e0141002. doi: 10.1371/journal.pone.0141002 (PMC4621059; doi:10.1371/journal.pone.0141002)
Supplement: S1 Protocol — (DOC) [file pone.0141002.s001.doc]

| **Improving efficacy and safety of TB and HIV Treatment by Nutritional Supplementation**  **Research protocol**  **January 2010**  **Participating Institutions**   1. Department of Infectious Disease, Rigshosptalet, University of Copenhagen, Denmark 2. National Institute for Medical Research, Tanzania 3. Department of Human Nutrition, Faculty of Life Sciences, University of Copenhagen, Denmark 4. Department of Internal Medicine, Jimma University Hospital, Ethiopia 5. Division of Clinical Pharmacology, University of Cape Town, South Africa 6. Department of Immunology, University of Bergen, Norway |
| --- |

# PARTICIPATING INSTITUTIONS AND COLLABORATORS

1. Department of Infectious Diseases, Rigshospitalet, University of Copenhagen

Åse Bengård Andersen, MD, DMSc, Consultant, Associate Professor: - Principal Investigator

Department of Infectious Diseases M5132, Rigshospitalet, Blegdamsvej 9, DK 2100 Copenhagen OE

2. National Institute for Medical Research (NIMR), Tanzania

2.1 Muhimbili Medical Research Centre, Box, 3436, Dar Es Salaam, Tanzania

Nyagosya S. Range, PhD; Principal Research Scientist, Local Principal Investigator

2.2 Mwanza Medical Research Centre, Box 1462, Mwanza Tanzania

John M, Changalucha, MSc. NIMR Mwanza Centre Director

Jeremiah Kidola, MD, MSc. Research Scientist, Study Coordinator (PhD candidate)

George PrayGod, MD, MPH, Research Scientist,

3. Department of Human Nutrition, Faculty of Life Sciences, University of Copenhagen, Denmark

Henrik Friis, MD, PhD, Professor of International Nutrition

Kim Fleicher Michaelsen, MD, PhD, Professor of International Nutrition

4. Department of Internal Medicine, Jimma University Hospital, Ethiopia

Alemeshet Yami, MD, Assistant Professor of Internal Medicine, Local – PI & PhD candidate

Department of Pediatric, Jimma University Hospital, Ethiopia

Tsinuel Girma, MD.

5. Division of Clinical Pharmacology, University of Cape Town, South Africa

Helen Mclleron, MD, PhD

Gary Maartens, MD, PhD, Professor, Head of Division

6. Department of Immunology, University of Bergen, Norway

Harleen Grewel, PhD, Professor of Microbiology

# FUNDING:

The study is funded by DANIDA (Danish International Development Agency) Grant No. **09-026RH.**

TABLE OF CONTENT

[PARTICIPATING INSTITUTIONS AND COLLABORATORS 1](#__RefHeading___Toc251667310)

[FUNDING: 1](#__RefHeading___Toc251667311)

[TABLE OF CONTENT 2](#__RefHeading___Toc251667312)

[SUMMARY: 3](#__RefHeading___Toc251667313)

[1.0 INTRODUCTION AND RATIONALE: 4](#__RefHeading___Toc251667314)

[2.0 OBJECTIVES: 5](#__RefHeading___Toc251667315)

[2.1 General objective: 5](#__RefHeading___Toc251667316)

[2.2 Specific objectives: 5](#__RefHeading___Toc251667317)

[2.3 Subsidiary objective: 5](#__RefHeading___Toc251667318)

[3.0 STUDY DESIGN AND METHODOLOGY: 6](#__RefHeading___Toc251667319)

[3.1 Study design: 6](#__RefHeading___Toc251667320)

[3.2 Study population: 6](#__RefHeading___Toc251667321)

[3.3 The interventions and study sites: 6](#__RefHeading___Toc251667322)

[3.4 Pharmacological Analyses: 7](#__RefHeading___Toc251667323)

[3.5 Filter-paper sampling: 8](#__RefHeading___Toc251667324)

[3.6 TB outcome: 8](#__RefHeading___Toc251667325)

[3.7 HIV related analyses: 8](#__RefHeading___Toc251667326)

[3.8 Anthropometric analyses and physical activity: 8](#__RefHeading___Toc251667327)

[4.0 EXPECTED OUTCOME: 9](#__RefHeading___Toc251667328)

[5.0 STATISTICAL ANALYSIS: 9](#__RefHeading___Toc251667329)

[6.0 RISKS AND ASSUMPTIONS: 9](#__RefHeading___Toc251667330)

[7.0 ETHICAL CONSIDERATIONS: 9](#__RefHeading___Toc251667331)

[7.1 Ethical approval: 9](#__RefHeading___Toc251667332)

[7.2 Enrolment of patients and informed consent: 9](#__RefHeading___Toc251667333)

[7.3 Incentives/compensation: 10](#__RefHeading___Toc251667334)

[8.0 CAPACITY BUILDING: 10](#__RefHeading___Toc251667335)

[9.0 DATA DISSEMINATION 10](#__RefHeading___Toc251667336)

[10.0 BUDGET: 10](#__RefHeading___Toc251667337)

[11.0 TIME LINE: 10](#__RefHeading___Toc251667338)

[12.0 INVESTIGATORS 10](#__RefHeading___Toc251667339)

[13.0 REFERENCES: 11](#__RefHeading___Toc251667340)

**Improving efficacy and safety of TB and HIV treatment by nutritional supplementation**

# SUMMARY:

Tuberculosis and HIV are two major health threats in Sub-Saharan Africa linked to poverty and malnutrition. This project is addressing the impact of simple and cheap, defined nutritional interventions during early treatment phases of both HIV and TB. The study sites are in Jimma, Ethiopia and in Mwanza, Tanzania. The main focus of the Ethiopian study will be on HIV; while in Tanzania it will be on TB and HIV.

The hypothesis is that nutritional supplements containing all micronutrients: vitamins and minerals as well as a protein energy source will increase drug absorption, ameliorate adverse effects and improve recovery of lean body mass.

HIV and TB treatment markers along with drug concentration profiles, information on body composition and adverse effects will be obtained from patients initiating on ART or TB treatment. In Ethiopia the nutritional intervention is a “Plumpy-Nut” based product composed of either whey or soy protein. In Tanzania, the intervention is a biscuit-format-product, also used in previously approved study in Mwanza[[1]](#footnote-2)*. The HIV patients will receive the intervention for three months either immediately at time of ART initiation or deferred. The TB patients (with or without HIV co-infection) will be randomised either to no intervention (= standard of care) or to receive the intervention during the initial intensive phase of TB treatment. Each TB patient will undergo two sessions of blood sampling for pharmacokinetic (PK) analyses: after steady state is obtained i.e. 5 to 9 days of treatment and later at the end of the intensive phase i.e. 2 months.

The outcome parameters will be: drug exposure (AUC = area under the curve) before and after nutritional intervention related to initial body composition and obtained weight gain, CD4 recovery and TB culture conversion rate. The frequency and severity of adverse effect will also be assessed.

The results may contribute to future management of TB and HIV patients in regions where under nutrition is prevalent.

The project is planned to run for three years (2010 through 2012) and the project is funded through the Danish “Consultative Research Committee for Development Research” = “DANIDA” through a grant including a budget for the Tanzanian project of 350 million TZs. The costly serum concentration analyses will be performed at Cape Town University in South Africa and the expenses for these analyses are covered through the Danish part of the budget.

# 1.0 INTRODUCTION AND RATIONALE:

Tuberculosis (TB) is a life threatening infectious disease linked to poverty, crowding and malnutrition. The global TB incidence curve is still increasing in Sub-Saharan Africa, due to the HIV pandemic, which hit hard in this region^1^. Because HIV impairs the cellular immune function, the progression of latent TB infection to active disease is accelerated from a lifetime risk of 5 -10 % to an **annual** risk of 10%. The signs and symptoms of advanced HIV infection is often unintended weight loss or extreme wasting mimicking disseminated TB disease and concomitantly HIV and TB infected patients are fulfilling the AIDS criteria.

The access to nutritious food in Sub-Saharan Africa is by no means guarantied and the disease associated loss of appetite, reduced physical strength and thereby reduced working capacity are just some aspects of the vicious circle these patients enter. The biological effects of under-nutrition are well known as increased co-morbidities and eventually mortality. Nevertheless, the evidence for nutritional supplementation to both HIV and TB infected patients is scarce. Two recent Cochrane articles have reviewed the literature on intervention studies and for both diseases the conclusions are clear: data is lacking^2;3^ .

The obvious need for further research and recommendations for action within the area of nutrition and the use of Anti Retroviral Therapy (ART) in resource-limited settings was also recently compiled in a WHO document from 2005^4^. These years, efforts are devoted to giving more patients in need access to ART. However, the drugs available for the African continent are no longer the drugs of choice in the western part of the world due to long-term metabolic adverse effects (especially by stavudine). It has been shown that the whole body protein turnover in both asymptomatic but even more so in symptomatic HIV patients is increased by up to 25% compared to HIV negative controls. However, only eight clinical trials have been identified addressing the effect of nutritional supplementation of HIV patients – and all of these were performed in high income countries (North America and Europe), the components of the interventions varied and no hard endpoints like death and/or co-morbidity were measured^3^. The weight loss caused by the HIV infection and the co morbidities is to a large extent lean body mass but the weight gains during recovery are mainly fat due to limited access to high quality, energy- and nutrient-dense supplements. This is believed to have a negative impact on the health and survival of the HIV infected population, posing the patients at increased risk for developing metabolic syndrome. It has become increasingly clear that the small intestine and the gut associated lymphoid tissue are severely damaged during acute but also chronic phase HIV^3^. The impact of this condition itself, concomitant diarrhoea and under-nutrition on ART drug absorption and hence effect is another important issue. Although the number of different HIV drug targets and drugs are growing there is a high degree of class resistance and costs still prevent 2^nd^ line ART to be generally available in most African countries.

The number of drugs available for treatment of TB is still very limited and only few drugs are in the development-pipeline. These years the world is witnessing an alarming increase in the number of multi-drug-resistant (MDR) cases of TB, and the need for new drugs was further stressed, when the reports from Kwazulu-Natal in 2006 reported of patients suffering from extremely-drug-resistant (XDR) strains causing fatal outcome in almost all HIV patients in a very short span of time ^1;5^. It is therefore important that the currently available drugs are administered correctly to avoid further development of treatment-resistant cases. A number of factors will influence the combined outcome for each case: e.g. patient adherence and quality of the drugs. However, co-morbidities like HIV, diabetes and malnutrition are also believed to play a role. The standard TB first-line drugs were developed and licensed decades ago – long before the era of HIV. Only few studies exist addressing the treatment outcome in patients with these co-morbidities ^6-10^. Already in 1984, a small study showed a reduced rifampicin drug exposure in a group of undernourished subjects^11^. Low anti-TB drug concentrations have been reported in a number of studies in HIV patients^6^ and cases of resistant TB caused by HIV related malabsorbtion have been reported^12^. Intermittent TB treatment in HIV patients, especially to those with low CD4 levels, is not recommended because of increased risk of relapse^13^. A study from Botswana found in a cohort of 91 patients that 89% had low serum concentrations of at least one TB drug ^7^, a finding in accordance with a pharmacokinetic study by our co-investigator of 142 TB patients in South Africa^14^. A study from Indonesia recently demonstrated that Rifampicin levels are strongly reduced in TB patients with type-2 diabetes^15^.

**In conclusion:**

HIV and TB control strategies in developing countries need to be robust and all possible low-cost assets, which may **ensure** and **improve the outcome of the current HIV and TB treatment regimen** should be enforced. We intend to assess the impact of nutritional status and the effect of **simple, cheap nutritional supplements** in the management of TB and HIV patients in two Sub Saharan settings.

# 2.0 OBJECTIVES:

## 2.1 General objective:

The main objective of the study is to improve efficacy and safety of ART and TB treatment in under-nourished patients in Sub-Saharan Africa.

## 2.2 Specific objectives:

1. To assess the effect of protein energy and micronutrient (vitamin/mineral) supplementation on safety and efficacy of ART and anti-TB treatment.

Specifically to assess the effect on energy and micronutrient supplementation on:

- - 1. TB drug levels in adult pulmonary TB patients with and without HIV
    2. Non-nucleoside reverse transcriptase inhibitor (NNRTI) and NRTI drug levels in an HIV population initiating treatment
    3. TB treatment markers: time to culture conversion in TB patients with and without HIV
    4. HIV treatment markers: HIV viral load decrease, CD4 count increase and HIV associated co-morbidities in an HIV population initiating ART
    5. Treatment related adverse effects, weight gain, body composition and physical activity in HIV patients initiating ART and pulmonary TB patients with and without HIV.

## 2.3 Subsidiary objective:

- - 1. immunological restoration (antigen specific cytokine profiles) during the intensive phase treatment of TB patients with or without HIV

# 3.0 STUDY DESIGN AND METHODOLOGY:

## 3.1 Study design:

Two clinical trials involving nutritional supplementation by two different products for a limited time period to HIV patients initiating ART and TB patients, with or without HIV, initiating TB treatment. The study will allow us to assess the effects on a number of well-defined outcome measures designed to reveal a potential beneficial or an indifferent effect. The project will form the basis of an African based research network by strengthening two Sub-Saharan study sites working on related projects, by capacity building both on direct patients care and by PhD training of two African candidates, and improve local laboratory facilities.

## 3.2 Study population:

The study will recruit 300 HIV positive patients referred for ART at Jimma University Hospital, Jimma, Ethiopia and 100 newly diagnosed pulmonary TB patients with or without HIV co-infection at Mwanza, Tanzania.

## 3.3 The interventions and study sites:

Two well defined nutritional interventions will be assessed in this study: Special composed high energy biscuits containing vitamins, minerals and protein produced by “Compact a/s” Aarhus, DK and a semisolid, ready to use supplementary food (RUSF) format “Plumpynut” produced especially for this project by “Nutriset”, France.


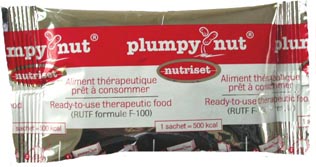


The “Plumpynut” format has been studied in paediatric populations by Médecins Sans Frontières (MSF) in Africa and proved widely accepted and operational. The acceptability in adults is less documented. Both products are low-cost interventions that may be produced locally.

- TB patients: The TB patients will receive either no intervention (standard of care) or receive 5 high-energy biscuits containing approximately 1000 kcal plus vitamins, minerals, zinc and selenium each day during the 2-month initial intensive treatment phase as part of the Directly Observed Treatment (DOT) regimen.
- HIV patients: will receive whey or soy based nutritional supplement covering approximately 50% of the daily energy need (1000 kcal) and all micronutrients including zinc and selenium for three months either at inclusion or deferred for three months. (Severely malnourished patients with a BMI < 16 kg/m^2^ will be excluded and treated according to National Guidelines for Treatment of severe acute malnutrition).

Structured interviews to assess **adherence**, **adverse effects** and **diet/additional food intake** will be part of the study.

**Jimma, Ethiopia:**

A randomised nutrition intervention trial among 300 HIV patients eligible for ART is planned to commence spring 2010. The trial is funded by the US Export Dairy Council through Department of Human Nutrition at the University of Copenhagen to compare a whey-based regimen with a soy-based intervention.

All patients will be followed for one year and assessed for effect on nutritional status (lean body mass, bioelectrical impedance and physical activity), adverse effects and HIV associated parameters: CD4 count, viral loads and co-morbidities. Based on this trial, we propose to measure serum levels of the NNRTIs (efavirens and nelfinavir) and NRTIs after 1 week and 3 months. (See time table). The clinic has currently around 1500 patients on ART and has been including 30 new HIV patients each month during the preceding 6 months. In a pilot study of 200 newly referred HIV patients showed that the mean BMI was only 19.2 (SD = 2.6) kg/m^2^ and 23 % were severely wasted i.e. a BMI less than 16 kg/m^2^.

**Mwanza, Tanzania:**

We have recently studied a cohort of adult (> 15 years) pulmonary TB patients in Mwanza. At the time of diagnosis the patients were in general malnourished with over 55% having a BMI < 18 kg/m^2^. We demonstrated in a randomised clinical trial that the supplementation of minerals and vitamins along with the anti-TB treatment led to a 2.3 kg greater weight gain irrespective of HIV status and further significantly reduced mortality in the HIV patients ^16-18^. This led to a second randomised, still ongoing trial among HIV infected TB patients assessing the role of energy and protein supplementation on top of micronutrient supplementation. Recruitment to this study has just been completed, reaching 1250 patients. The study is coordinated by the National Institute for Medical Research (NIMR) in Mwanza. The Danish National Committee on Biomedical Research Ethics ref. no. 2005-7041-57 and the local ethics committee approve the trial and it was registered as NCT 00311298 at the NIH clinical trials database.

The present proposal is an extension of this ongoing trial. TB patients will be recruited from Sekou Toure Regional Hospital, which is located within walking distance to the laboratory facility at NIMR. The TB diagnosis and TB treatment will be initiated within the framework of the National TB and Leprosy control Programme (NTLP) but samples for all patients will be send for confirmatory diagnosis including **culture** at the zonal TB reference laboratory (ZTRL) at Bugando Medical Centre (BMC) in Mwanza. Anti-TB drug resistance analyses will be performed at the Central TB Reference Laboratory (CTRL) which is situated at Muhimbili National Hospital (MNH) in Dar es Salaam. The recent establishment of a clinical trials unit has further strengthened the Mwanza site: the Mwanza Intervention Trials Unit (MITU) headed by Dr. Saidi Kapiga.

HIV positive (n=50) and HIV negative (n=50) pulmonary TB patients (PTB) will be randomised to receive high-energy protein and micronutrient containing biscuits or no nutritional intervention = standard of care treatment. The body composition, diabetes status and various para-clinical analyses of the patients will be assessed at entry and after 1 week, 2 and 6 months (see time table). The pharmacological analysis will be performed at study entry (after steady state is obtained after one week of treatment) and at the two-month visit. Data from the preceding years allow us to expect 50 patients to be included in each group for the first year. Only very few of the patients (less than 2.5%) are receiving concomitant ART at this time point (before 2-months) and they will not be included.

## 3.4 Pharmacological Analyses:

The blood samples will be collected and stored locally at –70^o^C. The majority of the pharmacological analyses will be performed at the University of Cape Town, Division of Pharmacology under the supervision of Drs Helen McIlleron and Gary Martens. The laboratory has extensive routine in analysing all conventional TB drugs, and the HIV drugs including the NNRTIs efavirens and nevirapine but also the NRTIs zidovudine, lamivudine and stavudine.

**HIV**: As mentioned above, NRTI and NNRTI drug levels will be measured at Cape Town laboratory and will require an extra blood sample of approximately 10 ml from each patient. We are also planning to set up an ELISA based method locally, which is working well at our laboratory at the Dept. of Infectious Disease, Rigshospitalet and has been successfully adapted to a Tanzanian setting under the supervision of Dr. T. Katzenstein^19^ and ARK Diagnostics, Freemont, CA. We expect – as reported from other studies - interpersonal drug level variations and therefore primarily intend to assess the change over time for each patient with or without nutritional substitution.

**TB:** The TB drugs of the day will be taken under observation while the patient is fasting. Blood samples will be drawn after 1, 2, and 6 hours in conjunction with the blood tests scheduled for the nutrition study. We expect interpersonal variation and will focus on the change over time for each patient receiving or not receiving nutritional supplementation with or without HIV co-infection. Rifampicin (RIF), isoniazid (INH), pyrazinamide (PZA), and ethambutol (EMB) levels and INH and RIF metabolites will be determined in all study participants from whole blood samples of approximately 10 ml. The samples will be kept on wet ice for a maximum of 1 hour before processing by centrifugation and storage at –70^o^C. Patient samples and samples of the drug product of the batch being used will be transferred to Cape Town, SA. Population based nonlinear mixed effects modeling will be used to describe structural population models describing the C_max_ of the anti TB-drugs and their metabolites and to quantify the effects of patient (body composition, hemoglobin, white blood cell and CD4 counts) and drug formulation (batch, time to expiry) covariates on the model.

## 3.5 Filter-paper sampling:

The Cape Town group are working on methods to perform PK analyses on whole blood samples, dried directly on filter paper. We intend to recover parallel filter paper samples for further studies on this methodological aspect, which eventually would be very useful for studies in resource poor settings.

## 3.6 TB outcome:

Most TB studies from developing countries rely on microscopy of acid-fast bacilli (AFB) as an outcome parameter. Especially in the initiation phase of treatment it is important to distinguish between live and dead bacteria (which is not possible by microscopy). Our access to the the Zonal reference laboratory for close monitoring of culture conversion is one of the strengths of this study site. The NIMR site is in a process of establishing bio safety facilities for culture of TB. The current project will strengthen this process and allow handling of patient samples at NIMR also. Samples for later immunological analyses will be processed at NIMR and stored at -70oC for later analyses in Bergen (Professor Harleen Grewal).

## 3.7 HIV related analyses:

We have access to CD4 quantisation locally both in Jimma and at NIMR, Mwanza. Quantitative HIV RNA analyses will be performed at the Ethiopian Health and Nutrition Institute in Addis Ababa HIV and locally at the NIMR, Mwanza. Other para-clinical parameters are also being analysed locally.

## 3.8 Anthropometric analyses and physical activity:

The body composition will be assessed at base line and at follow-up visits according to the time table shown below. The parameters assessed include; height/weight/body mass index (BMI) assessment, hip-waist ratio, triceps skinfolds (TSF) and middle upper arm circumference (MUAC) and bioimpedance. The physical activity will be assessed by grip strength measurements and “Acti-heart” monitoring (A small, portable accelerometer and heart-rate-monitor worn by the test person for 48 hours) – only performed in Jimma, Ethiopia.

# 4.0 EXPECTED OUTCOME:

If the nutritional intervention has a positive effect on drug concentration levels and HIV related surrogate markers and a negative effect on the number and severity of adverse effects and co-morbidities the results may be used as support for a change in management of newly diagnosed TB/HIV patients. A nutritional supplement in the format of an energy-dense and micronutrient containing biscuit of RUSF is a low-cost, easy to implement, initiative which may improve HIV and TB treatment of the poor. Future local production of RUSF will strengthen local infrastructure by creating jobs and opportunities for small-scale local enterprises.

# 5.0 STATISTICAL ANALYSIS:

Data will be double entered in EpiData and will be analysed using the STATA software program.

The difference in adherence between the two groups will is assessed by Pearson’s χ2 test with the use of robust standard errors. Logistic regression analysis will be used to determine factors associated with adherence to treatment. In order to ensure that analyses are not driven by results, but rather by the pre-defined research questions; all statistical analyses will be carried out according to the statistical analysis plan that is drawn up before the end of data collection.

**Null hypothesis:** The null hypothesis is that there will be no detectable difference in the ART or anti-TB drug levels (AUC) in the groups receiving nutritional supplementation compared to those not receiving – or receiving it at deferred time points. There will be no differences in culture conversion of TB patients at 2 months and the CD4 increase and decline in HIV RNA levels will not be affected. The physical strength and activity will be the same in those receiving and those not receiving nutritional supplementation.

**Detectable differences**: We will be able to detect an increase of 44 CD4 cells per μl if we include 100 HIV patients in each group (250 patients are planned compared to 100 in the deferred group). We will be able to detect an increase in lean body mass of 1.65 kg if enrolling 100 patients with a mean lean body mass of 40 kg (SD = 4.2).

# 6.0 RISKS AND ASSUMPTIONS:

Inclusion of too few patients: Due to our yearlong collaboration with NIMR, Mwanza we do not expect lack of patients to be a problem. Our collaborators in Jimma are very dedicated and a “letter of understanding” between the University of Jimma and the University of Copenhagen, Department of Human Nutrition supports the genuine collateral involvement in the project. This has been further reinforced by preparatory visits by our Ethiopian colleagues in Denmark and pilot studies in Jimma.

# 7.0 ETHICAL CONSIDERATIONS:

## 7.1 Ethical approval:

Both studies will be submitted for approval by local ethical committees and Danish Central Ethical Committee. Both studies will be filed at the NIH clinical trials database. Study progress reports will be submitted to the National Medical Research Coordinating Committee (MRCC).

## 7.2 Enrolment of patients and informed consent:

Only patients giving informed consent will be included in the study. Patients will be free to withdraw from the study at any time during the intervention. Withdrawal from the study will not interfere with standard of care and treatment of the patient.

## 7.3 Incentives/compensation:

The TB patients accepting serial blood sampling for PK analyses will receive reimbursement of transport expenses and lost income. In addition refreshments will be provided during the waiting hours at the clinic. All TB patients will be treated according to the Tanzanian standard TB regimen. HIV positive patients will be referred to CTC for care and patients with raised p-glucose (as described in detail in the SOPs) will be referred to local diabetes clinic for management.

# 8.0 CAPACITY BUILDING:

**Postgraduate training**: Two projects at PhD level will be initiated through the research involved in the present project (one in each country) and potential candidates have already been identified. The Weill Bugando University College of Health Sciences in Mwanza is currently launching a PhD program and we intend to submit a PhD project proposal to this institution for the Tanzanian candidate.

9.0 DATA DISSEMINATION:

Results from the study will be communicated and presented at local and international meetings and conferences. We intend to publish the results in international, peer reviewed scientific journals which will comprise the major component of the planned PhD project..

# 10.0 BUDGET:

The present proposal was accepted and a final letter of grant was received in December 2009

(Grant No. 09-026RH).

The three-year grant for the Tanzanian part of the project is DKK 1,261,041 (~ Tshs: 350.000.000).

This amount is expected to cover costs for the project implementation including; transport (car maintenance and fuel), allowances and salaries for study personnel, materials (equipment and supplies), stipend for PhD candidate and local project supervision travels. Institutional overhead (7%) is also included. The pharmacological analyses performed in Cape Town will be covered from the Danish part of the grant. An amount allowing 10 months stay in Denmark for the PhD candidate for write up of the PhD thesis is included in the Danish budget.

# 11.0 TIME LINE:

The study is planned to last for three years 2010-2012. Implementation is expected to start in April 2010 (see attached).

12.0 INVESTIGATORS**:**

**Principal Investigator**: Åse Bengård Andersen (Sund., Univ. of Cph.),

**Local Co-PIs**: Nyagosya Range (NIMR, Tanzania) and Alemeshet Yami (Jimma, Ethiopia)

**Study coordinator**: Jeremiah Kidola (Mwanza, Tanzania).

**Collaborators:** Henrik Friis, Kim Fleicher Michaelsen, and Pernille Kæstel (Life, Univ. of Cph.), George Praygod, John Changalucha, and Saidi Kapiga (NIMR and MITU, Tanzania), Helen McIlleron and Gary Martens (SA), Harleen Grewal (Univ. of Bergen), and Tsinuel Girma (Jimma, Ethiopia).

# 13.0 REFERENCES:

1. Global Tuberculosis Control, Surveillance, Planning, Financing. WHO Report 2007.

2. Abba K, Sudarsanam TD, Grobler L, Volmink J. Nutritional supplements for people being treated for active tuberculosis. Cochrane Database Syst Rev 2008 ;(4):CD006086.

3. Mahlungulu S, Grobler LA, Visser ME, Volmink J. Nutritional interventions for reducing morbidity and mortality in people with HIV. Cochrane Database Syst Rev 2007;(3):CD004536.

4. Raiten DJ, Grinspoon S, Arpadi S. Nutritional considerations in the use of ART in resource-limited settings. World Health Organization. 2005.

5. Gandhi NR, Moll A, Sturm AW et al. Extensively drug-resistant tuberculosis as a cause of death in patients co-infected with tuberculosis and HIV in a rural area of South Africa. Lancet 2006; 368(9547):1575-1580.

6. Gurumurthy P, Ramachandran G, Hemanth Kumar AK et al. Malabsorption of rifampin and isoniazid in HIV-infected patients with and without tuberculosis. Clin Infect Dis 2004; 38(2):280-283.

7. Tappero JW, Bradford WZ, Agerton TB et al. Serum concentrations of antimycobacterial drugs in patients with pulmonary tuberculosis in Botswana. Clin Infect Dis 2005; 41(4):461-469.

8. Perlman DC, Segal Y, Rosenkranz S et al. The clinical pharmacokinetics of rifampin and ethambutol in HIV-infected persons with tuberculosis. Clin Infect Dis 2005; 41(11):1638-1647.

9. Perlman DC, Segal Y, Rosenkranz S et al. The clinical pharmacokinetics of pyrazinamide in HIV-infected persons with tuberculosis. Clin Infect Dis 2004; 38(4):556-564.

10. Zhu M, Burman WJ, Starke JR et al. Pharmacokinetics of ethambutol in children and adults with tuberculosis. Int J Tuberc Lung Dis 2004; 8(11):1360-1367.

11. Polasa K, Murthy KJ, Krishnaswamy K. Rifampicin kinetics in undernutrition. Br J Clin Pharmacol 1984; 17(4):481-484.

12. Patel KB, Belmonte R, Crowe HM. Drug malabsorption and resistant tuberculosis in HIV-infected patients. N Engl J Med 1995; 332(5):336-337.

13. Pozniak AL, Miller RF, Lipman MC et al. BHIVA treatment guidelines for tuberculosis TB/HIV infection 2005. HIV Med 2005; 6 Suppl 2:62-83.

14. McIlleron H, Wash P, Burger A et al. Determinants of rifampin, isoniazid, pyrazinamide, and ethambutol pharmacokinetics in a cohort of tuberculosis patients. Antimicrob Agents Chemother 2006; 50(4):1170-1177.

15. Nijland HM, Ruslami R, Stalenhoef JE et al. Exposure to rifampicin is strongly reduced in patients with tuberculosis and type 2 diabetes. Clin Infect Dis 2006; 43(7):848-854.

16. Range N, Magnussen P, Mugomela A et al. HIV and parasitic co-infections in tuberculosis patients: a cross-sectional study in Mwanza, Tanzania. Ann Trop Med Parasitol 2007; 101(4):343-351.

17. Range N, Changalucha J, Krarup H et al. The effect of multi-vitamin/mineral supplementation on mortality during treatment of pulmonary tuberculosis: a randomised two-by-two factorial trial in Mwanza, Tanzania. Br J Nutr 2006; 95(4):762-770.

18. Range N, Andersen AB, Magnussen P, Mugomela A, Friis H. The effect of micronutrient supplementation on treatment outcome in patients with pulmonary tuberculosis: a randomized controlled trial in Mwanza, Tanzania. Trop Med Int Health 2005; 10(9):826-832.

19. Holland D, Espina-Quinto R, Stefanski E, Moon B, Valdez J, Connor J. Rapid automated immunoassay for TDM of nevirapine using ARK NVP-Test: Method validation, application and comparison with HPLC method. abstract, CROI 2007 .

1. ^^ The role of nutritional support and diabetes during treatment of pulmonary tuberculosis:

   two randomized nutritional supplementation trials in Tanzania (2006 – 2008) [↑](#footnote-ref-2)
